# Assembly chaperone Nas6 selectively destabilizes 26S proteasomes with defective regulatory particle-core particle interfaces

**DOI:** 10.1016/j.jbc.2023.102894

**Published:** 2023-01-10

**Authors:** Jennifer L. Warnock, Gabriel W. Jobin, Sandhya Kumar, Robert J. Tomko

**Affiliations:** Department of Biomedical Sciences, Florida State University College of Medicine, Tallahassee, Florida, USA

**Keywords:** 26S proteasome, chaperone, quality control, ubiquitin, proteolysis, assembly, macromolecular complex, CP, core particle, rNas6, recombinant Nas6, RP, regulatory particle, RP_1_CP, singly-capped proteasome, RP_2_CP, doubly-capped proteasome

## Abstract

The 26S proteasome is a 66-subunit–chambered protease present in all eukaryotes that maintains organismal health by degrading unneeded or defective proteins. Defects in proteasome function or assembly are known to contribute to the development of various cancers, neurodegeneration, and diabetes. During proteasome biogenesis, a family of evolutionarily conserved chaperones assembles a hexameric ring of AAA+ family ATPase subunits contained within the proteasomal regulatory particle (RP) and guide their docking onto the surface of the proteolytic core particle (CP). This RP–CP interaction couples the substrate capture and unfolding process to proteolysis. We previously reported a mutation in the proteasome that promoted dissociation of the RP and CP by one of these chaperones, Nas6. However, the nature of the signal for Nas6-dependent proteasome disassembly and the generality of this postassembly proteasome quality control function for Nas6 remain unknown. Here, we use structure-guided mutagenesis and *in vitro* proteasome disassembly assays to demonstrate that Nas6 more broadly destabilizes 26S proteasomes with a defective RP-CP interface. We show that Nas6 can promote dissociation of mature proteasomes into RP and CP in cells harboring defects on either side of the RP-CP interface. This function is unique to Nas6 and independent from other known RP assembly chaperones. Further biochemical experiments suggest that Nas6 may exploit a weakened RP-CP interface to dissociate the RP from the CP. We propose that this postassembly role of Nas6 may fulfill a quality control function in cells by promoting the recycling of functional subcomplexes contained within defective proteasomes.

The 26S proteasome (hereafter proteasome) is the endpoint of the ubiquitin-proteasome system and is responsible for the majority of regulated protein degradation in eukaryotic cells. Protein substrates destined for degradation are typically first modified with a chain of the small protein ubiquitin, which acts as a signal for delivery of the substrate to the proteasome. The proteasome captures the substrate *via* the polyubiquitin chain, unfolds it, and cleaves it into short peptides for recycling. Importantly, alterations to proteasomal assembly, structure, or function are linked to numerous human diseases (1, 2). Thus, there is considerable interest in exploiting the biology of the proteasome for pharmacologic therapy.

The proteasome is comprised of two main complexes: the regulatory particle (RP) and the core particle (CP). The RP can be further divided into lid and base subcomplexes. The lid consists of nine subunits: Rpn3, 5-9, 11, 12, and Rpn15/Sem1. The base consists of non-ATPase subunits Rpn1, Rpn2, and Rpn13, as well as a heterohexameric ring of AAA+ ATPases (3), Rpt1-6. During catalysis, the deubiquitinase subunit Rpn11 removes the polyubiquitin chain from the substrate. The ATPase ring interfaces directly with the ends of the barrel-shaped CP and converts chemical energy from ATP into mechanical force to unfold substrates and translocate them through a gated pore into the CP. The CP is comprised of two stacked heteroheptameric β rings formed from subunits β1-7, sandwiched between two heteroheptameric α rings formed from subunits α1-7. The α rings directly interface with the RP and also form a gated entryway into the peptidase sites within the β rings. The β1, β2, and β5 subunits display caspase-like, trypsin-like, and chymotrypsin-like peptidase activities, respectively (3, 4, 5), that cleave the protein substrate into short peptides.

The RP interfaces with the CP primarily *via* insertion of the flexible C-termini of selected Rpt ATPase subunits into pockets in the α-ring surface formed at the interfaces between adjacent subunits. The C-terminal tails of the Rpt subunits are highly conserved (6) and serve critical roles in proteasome biogenesis, in tethering the RP to the CP, and in regulating substrate access to the peptidase center of the proteasome (7, 8, 9, 10). Of the six ATPase subunits, the C-terminal tails of five ATPases have been observed to dock into the surface of the CP (7, 9, 11, 12). The C-termini of Rpt2, Rpt3, and Rpt5 contain highly conserved HbYX motifs (where Hb, Y, and X indicate a hydrophobic amino acid, a tyrosine, and any amino acid, respectively) that dock into the surface of the α-ring. Importantly, these three tails appear docked in all available structures of the 26S proteasome (7, 11, 12). In contrast, the Rpt1 and Rpt6 tails appear stably docked only in the structures of proteasomes that are chemically or genetically locked into an active substrate-processing state. Biochemical and genetic experiments have demonstrated that the passageway into the CP, formed by the N-termini of selected α-subunits, is fully opened only upon insertion of the Rpt1 and Rpt6 tails (7), leading to a model in which docking of the Rpt1 and Rpt6 C-termini primes the CP to accept incoming substrate as it is translocated into the CP by the ATPases.

Proteasome biogenesis requires an enormous resource commitment by the cell and is critically dependent on numerous intrinsic and extrinsic factors to ensure rapid and faithful assembly *in vivo* (13, 14). Among the extrinsic factors are a number of dedicated assembly chaperones that help to coordinate particular assembly steps. Although the roles of many assembly chaperones are still being investigated, their functions include stabilizing assembly intermediates prior to their incorporation into higher-order species, restricting inappropriate subunit incorporations, and matching the conformational states of particular intermediates to ensure they mesh properly during biogenesis (13, 15, 16). In healthy cells, four dedicated, evolutionarily conserved chaperones aid in RP assembly: Nas6, Rpn14, Nas2, and Hsm3 (p28/gankyrin, PAAF1, p27, and S5b, respectively in mammals). Each binds to a C-terminal region of a specific Rpt subunit to regulate RP assembly (13). Upon completion of their respective functions in proteasome biogenesis, these chaperones dissociate, yielding mature 26S proteasomes and recycling these chaperones for additional rounds of assembly.

Although multiple proteasome subunit assembly sequences may occur *in vivo* (17, 18, 19, 20, 21, 22, 23), most data support the presence of a preferred pathway that is followed under normal conditions, with some limited evidence that alternative pathways or mediators may be invoked under stress conditions (24). In the preferred assembly sequence, the CP, base, and lid subcomplexes each assemble independently, followed by joining of the lid and base to make the RP and culminating in RP-CP association to yield 26S proteasomes. One of the most intricate assembly processes is that for the base. Most studies indicate that assembly of the base proceeds *via* formation of three distinct chaperone-bound intermediates: Rpt4-Rpt5-Nas2, Hsm3-Rpt1-Rpt2-Rpn1, and Nas6-Rpt3-Rpt6-Rpn14-Rpn2-Rpn13. These three intermediates then associate stepwise to form the base (13, 14). One chaperone, Nas2, is released immediately prior to or upon completion of the base, whereas Nas6, Rpn14, and Hsm3 remain bound. The chaperone-bound base is next assembled with the lid subcomplex and an intrinsic proteasomal ubiquitin receptor subunit, Rpn10, joins and stabilizes the base–lid interaction (14, 25), yielding a chaperone-bound RP. This chaperone-bound RP can then associate with the CP, yielding mature proteasomes and evicting Nas6, Rpn14, and Hsm3 in the process. The exact mechanisms of chaperone eviction from nascent proteasomes are not yet understood but are likely coupled to conformational changes that occur during or immediately after RP and CP association (16).

At present, two pathways are known that mediate quality control of proteasomes after assembly has been completed. The first is mediated by a proteasome-interacting protein, Ecm29, which was reported to bind and either disassemble and repair (26, 27, 28) or inactivate (29, 30) defective proteasomes. In this pathway, phosphorylation of key sites on the C-terminus of the α7 subunit serves as a recruitment signal for Ecm29 to proteasomes, harboring defects in the Rpt5 subunit (26). A second pathway has been characterized by several groups, in which damaged or unneeded proteasomes can be removed from the cell *via* autophagy (14, 31, 32). Although some of the mediators for damaged *versus* unneeded proteasomes appear to differ (14, 31, 32, 33), both are eventually delivered to the vacuole/lysosome for destruction.

Recently, we reported a mutant of the RP lid subunit Rpn5 that caused a conformational defect in the RP (16). This mutant displayed a reduced abundance of mature, RP-capped proteasomes and an accumulation of free CP and RP, consistent with a weakened RP-CP interface. Importantly, the RP in this mutant was found to be bound by Nas6, but not other RP-associated chaperones, invoking a role for Nas6 in the process. Deletion of *NAS6* fully suppressed this phenotype, and it was exacerbated by *NAS6* overexpression, suggesting a possible quality control function for Nas6. Here, we show that Nas6 more broadly destabilizes mature 26S proteasomes harboring defects at the interface between the RP and CP. We find that this function is fulfilled uniquely by Nas6 and without assistance by other proteasome assembly chaperones. We provide evidence that Nas6 exploits the relative affinity difference between the RP and CP that occurs when their interface is damaged and that show that reinforcement of the RP-CP interface can bypass Nas6-dependent dissociation. We posit that Nas6 may fulfill a new facet of proteasome quality control, in which proteasomes with defective RP-CP interfaces are disassembled to permit recycling of functional subcomplexes into active proteasomes.

## Results

### A frameshift mutant altering the Rpt3 C-terminal tail leads to proteasome structural and functional defects

**Figure 1:**
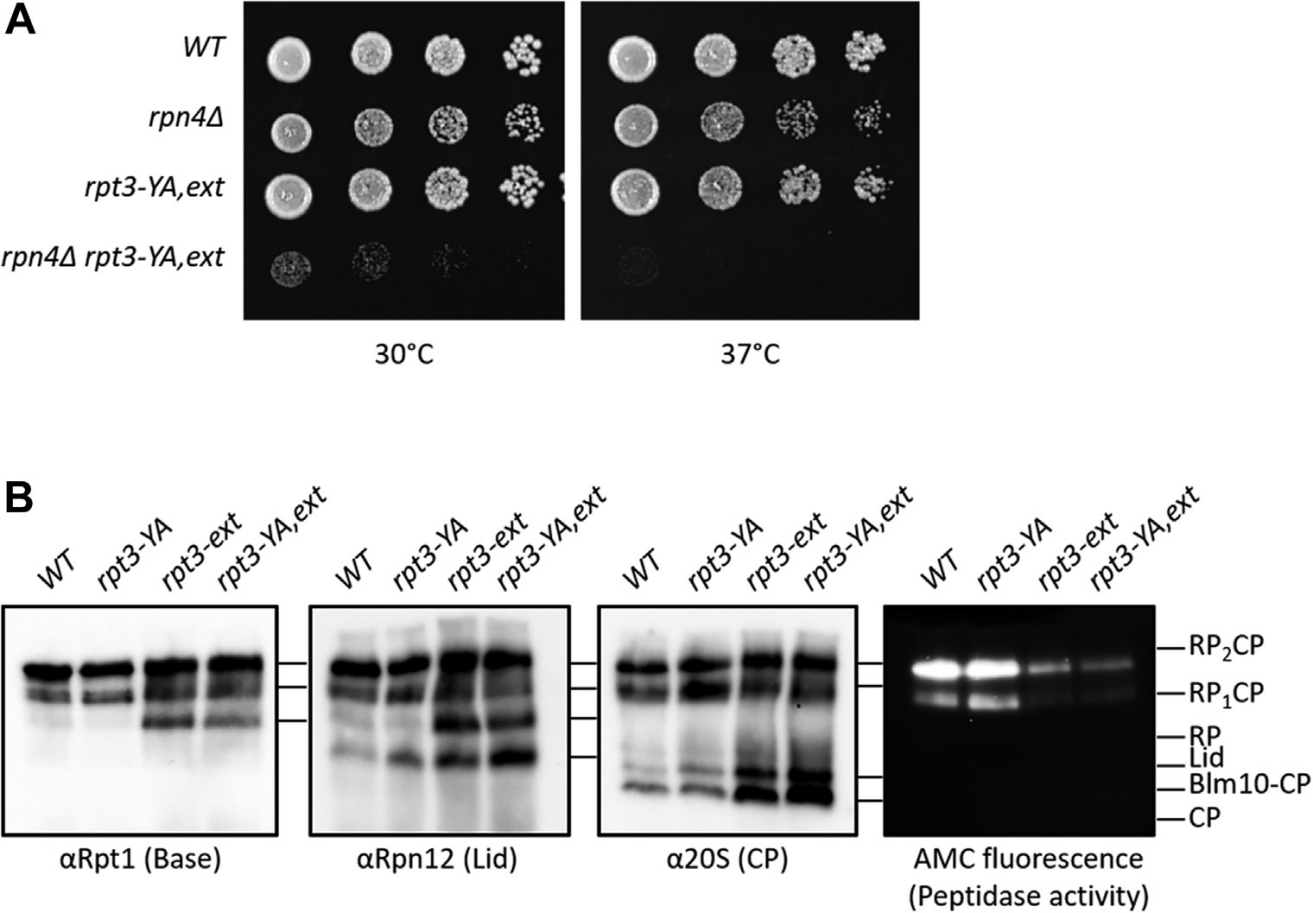
**A frameshift mutant altering the Rpt3 C-terminal tail leads to proteasome structural and functional defects.***A*, growth assay analysis of Rpt3 frameshift mutant (*rpt3-YA,ext*), which results in an extension of the Rpt3 C-terminal tail, with deletion of *RPN4*. Serial dilutions of indicated strains were plated on YPD media and incubated at indicated temperatures for 2 days. Growth of the double mutant is extremely sick at normal growing temperatures and lethal at elevated temperatures. *B*, native PAGE analysis of the panel of Rpt3 frameshift mutant (*rpt3-YA,ext*) and genetic separation of *rpt3-ext* and *rpt3-YA* mutants. Structural defects characterized by an accumulation of free RP, Lid, and CP observed in the frameshift mutant is due to the extension of the Rpt3 tail. Also shown is an in-gel peptidase assay showing a peptidase defect characterized by severely lower AMC fluorescence. The peptidase defect of the frameshift mutant is also due to extension of the Rpt3 tail. AMC, 7-amino-4-methylcoumarin; CP, core particle; RP, regulatory particle.

While investigating the function of the base ATPase ring in yeast, we discovered an unexpectedly strong phenotype linked to a mutant allele of *RPT3* encoding a substitution of its pore-1 loop tyrosine with alanine (34, 35, 36). In yeast, the fitness defects of proteasome hypomorphs can often be suppressed by compensatory transcriptional upregulation of new proteasome biogenesis. This compensatory upregulation is mediated by the transcription factor Rpn4 (37, 38). We assessed how the deletion of *RPN4* affected the cellular health and integrity of proteasomes when combined with the *rpt3-YA* allele and found that the combination led to severe growth defects, even at normal growth temperature (Fig. 1*A*), which was not observed in previous studies (34, 39).

At present, no structural role is known for the pore loops of the ATPase subunits, so this mutation was not anticipated to impact proteasome assembly or structure. We considered the possibility that the Rpt3 pore-1 loop influenced the RP-CP interface; however, recreation of the *rpt3-YA* allele *de novo* in a formerly WT strain failed to completely recapitulate the observed phenotype. This prompted us to resequence the original *rpt3-YA* allele. Upon sequencing, we unexpectedly discovered a spontaneous frameshift mutation that altered the length and sequence of the C-terminus of Rpt3 in addition to the expected tyrosine substitution in the pore loop. We renamed this pore and frameshift double mutant allele *rpt3-YA,ext*.

One of the most prominent biochemical effects of the *rpt3-YA,ext* allele was substantially reduced abundance of singly and doubly RP-capped proteasomes (RP_1_CP and RP_2_CP, respectively) when assayed by native PAGE and immunoblotting (Fig 1*B*, RP_2_CP and RP_1_CP), suggesting a structural defect. This loss of full proteasomes was accompanied by an accumulation of free RP and CP (Fig. 1*B*). Genetic segregation of the pore loop mutation (*rpt3-YA*) and the frameshift mutation revealed the RP-CP association defect was primarily due to the frameshift mutation (Fig. 1*B*). We designated the frameshift mutant allele, which encodes a WT pore loop, *rpt3-ext*.

### Disruption of Rpt3 tail docking into the CP promotes an RP-CP interaction defect

**Figure 2:**
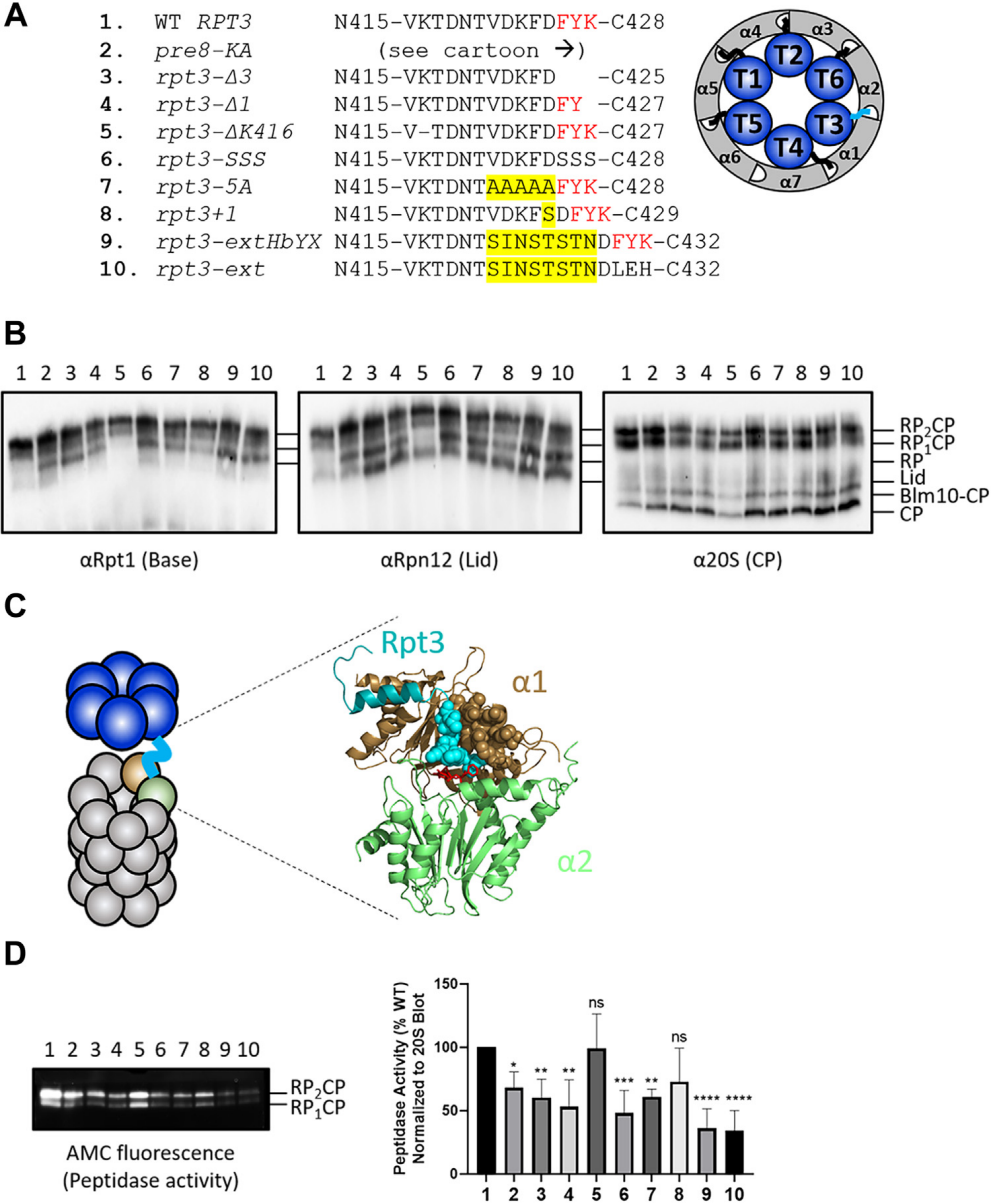
**Disruption of the Rpt3 tail docking into the CP promotes an RP–CP interaction defect.***A*, overview of Rpt3 C-terminal tail docking mutants. In *red* is the conserved HbYX motif. Highlighted are introduced mutations upstream of the HbYX motif. (*Right*) Cartoon diagram of the Rpt tails of the base docking into the pockets formed between CP α subunits. Shown in *red*, the Rpt3 tail docking into CP α2 (*PRE8*) pocket. *B*, native PAGE immunoblotting of the mutants shown in (*A*). *C*, cartoon (*left*) and atomic structure (*right*; PDB 6FVY) of the RP base (*blue*) docking onto the CP (*gray*). The Rpt3 tail (*cyan*) docks into the CP in the pocket formed by the α1 (*brown*) and the α2 (*green*) subunits. Spheres highlighting close contacts (≥4 Å) formed between the Rpt3 tail and the α1 subunit. HbYX motif shown in *red*. Other base and CP subunits as well as the N-terminal region of Rpt3 are omitted for clarity. *D*, in-gel peptidase assay of the panel of Rpt3 tail docking mutants (numbered as in *A*). Rpt3 tail docking mutants show a decrease in overall peptidase activity, indicated by lower AMC fluorescence. Quantification of proteasomal peptidase activity. Peptidase activity was normalized to total RP_2_CP and RP_1_CP abundances (*B*; α20S), with WT peptidase activity then set to 100%. One-way ANOVA with Tukey’s test for multiple comparisons; error bars represent ± SD; ns, not significant; ∗*p* < 0.05; ∗∗*p* < 0.01; ∗∗∗*p* < 0.001; ∗∗∗∗*p* < 0.0001; *N* = 5. AMC, 7-amino-4-methylcoumarin; CP, core particle; RP, regulatory particle.

The protein product of the *rpt3-ext* allele harbors nonconservative substitutions of the last eight amino acids in Rpt3, as well as an additional four amino acid extension (Fig. 2*A*). These substitutions include replacement of the HbYX motif found at the extreme C-terminus of the protein. Seminal work by others has shown that the HbYX motifs of Rpt2, Rpt3, and Rpt5 stably dock the RP onto the CP *via* insertion of these conserved motifs into pockets formed at the interfaces of selected CP α subunits (11, 12). Although truncation of the terminal amino acid of Rpt3 compromises activation of the CP (40), very little information exists on the importance of the amino acid sequences flanking the HbYX motif nor is it known whether the spacing of the HbYX motif relative to the globular body of Rpt3 influences its functions in RP-CP docking and CP gating.

To better understand how the environment near the Rpt3 HbYX motif contributed to proteasome structure and function, we generated a number of yeast strains harboring *RPT3* mutant alleles with varied C-terminal tail lengths, tail sequences, and HbYX motif integrities (Fig. 2*A*). These included a complete truncation of the HbYX motif (*rpt3-Δ3*), mutation of the HbYX motif to ser-ser-ser (*rpt3-SSS*), a previously reported truncation of the C-terminal lysine residue (*rpt3-Δ1*^10, 40^), deletion of a residue within the tail located highly distal to the HbYX motif (*rpt3-ΔK416*), insertion of one (*rpt3+1*) residue immediately prior to the HbYX motif, or mutation of the five amino acids preceding the HbYX motif to alanine (*rpt3-5A*). We also substituted the last three amino acids of the *rpt3-ext* mutant for phe-tyr-lys, the native HbYX motif of Rpt3 (*rpt3-extHbYX*). Finally, we generated a strain harboring a mutation in the pocket lysine (6) of α2 (encoded by the *PRE8* gene) that interacts with the HbYX motif of Rpt3 to mediate stable tail docking (Fig. 2*A*, right cartoon). This mutation leaves the tail of Rpt3 conveniently intact while disrupting stable tail docking into the CP. We then assessed the integrity of 26S proteasomes in each of these strains by native PAGE and immunoblotting.

As observed for our original frameshift mutant, proteasomes from the *rpt3-ext* mutant had a structural defect, evident as an accumulation of free RP, free CP, and free lid (Fig. 2*B*, lane 10). The accumulation of intact RP and CP suggested that the interface between these particles was weakened (we note free lid often accumulates in base mutants and is likely an indirect effect of these mutations (40)). Further, we observed a highly similar defect in *pre8-KA* cell extracts (Fig. 2*B*, lane 2), although a lower accumulation of free lid subcomplex was evident. This may reflect either a slightly less deleterious impact than other mutants, leading to less compensatory synthesis of new proteasome components, or potentially an impact of Rpt3 tail mutations on lid-base association that is not caused by the *pre8-KA* mutant. Nevertheless, the observation that this CP pocket mutant displayed similar phenotypes as the *rpt3-ext* mutant suggests that the accumulation of RP and CP is largely due to disruption of the Rpt3 tail docking into the CP. Substituting the native Rpt3 HbYX motif for the last three amino acids of *rpt3-ext* (*rpt3-extHbYX*) completely failed to rescue this defect (Fig. 2*B*, lane 9), indicating that either the tail length, sequence, or both were also important for normal docking of the Rpt3 HbYX motif. Similarly, truncating the HbYX motif partially (*rpt3-Δ1*) or entirely (*rpt3-Δ3*) as well as mutating the HbYX motif (*rpt3-SSS*) caused a defect of similar magnitude to the *rpt3-ext* mutation (Fig. 2*B*, lanes 4, 3, and 6, respectively). Together, these observations suggest that both the integrity of the N-terminal residues flanking the HbYX motif as well as the spacing of the HbYX motif from the body of Rpt3, are important for normal Rpt3 tail docking.

Intriguingly, mutation of the five amino acids prior to the HbYX motif to alanine (*rpt3-5A*) caused a structural defect indistinguishable from the *rpt3-ext* allele, despite maintaining WT spacing of the HbYX motif from the body of Rpt3 (Fig. 2*B*, lane 7). Inspection of available structures of the 26S proteasome indicate that these amino acids coil on top of the CP α-subunit pocket formed by α1 and α2, making substantial contact with the α1 subunit (Fig. 2*C*). In contrast, deletion of a lysine in an unstructured region of the Rpt3 tail that does not contact the CP or the Rpt3 globular body had no appreciable effect on proteasome integrity compared to WT, indicating that some shortening of the Rpt3 tail is tolerated as long as the residues that form contacts with the α1 and α2 subunits are not disrupted (Fig. 2*B*, lane 5). Complementary to the data presented here, we observed that combination of several Rpt3 tail docking mutants with deletion of *RPN4* led to growth defects observed both at normal and elevated temperatures (Fig. S1*A*). Together, these data show that disruption to the sequence of the Rpt3 tail, either in the conserved HbYX motif or directly upstream of the HbYX motif, leads to an RP-CP interface defect.

In the simpler, homomeric ATPase rings of Archaeal proteasomes, the HbYX motif also stimulates opening of a gated pore in the surface of the CP α ring, enhancing substrate access to the proteolytic chamber and thereby stimulating proteolysis. In eukaryotic proteasomes, the terminal amino acid of Rpt3 must be present for efficient gating of the CP (10, 40). Examining the activity of RP-capped proteasomes *via* an in-gel peptidase assay revealed a close correlation between the severity of the structural defect and the peptidase activity. Importantly, when the measured peptidase activity was normalized to the abundance of doubly and singly capped proteasomes, we observed a significant reduction in the peptidase activity of each *RPT3* mutant displaying a structural defect (Fig. 2*D*). Together, this indicates that docking of the Rpt3 tail is necessary for proper gating of the CP, consistent with previous reports.

The structural and functional defects that precipitated from these various Rpt3 mutations were not due to the altered expression of Rpt3 (Fig. S1*B*) or activity of the CP (Fig. S1*C*, red box), and we were able to rescue the structural and functional defects in a representative Rpt3 mutant, *rpt3-SSS*, by ectopically expressing a WT copy of *RPT3* (Fig. S1*D*). In contrast, expression of *RPT3* in the *pre8-KA* mutant did not, supporting a specific role for the Rpt3 tail. In sum, stable docking into and activation of the CP by the Rpt3 tail is dependent on an intact HbYX motif, flanking residues upstream of the HbYX motif, and on the proper spacing of the HbYX motif from the body of the Rpt3 subunit.

### RP assembly chaperone Nas6 associates with free RP in mutants with weakened RP-CP interfaces

**Figure 3:**
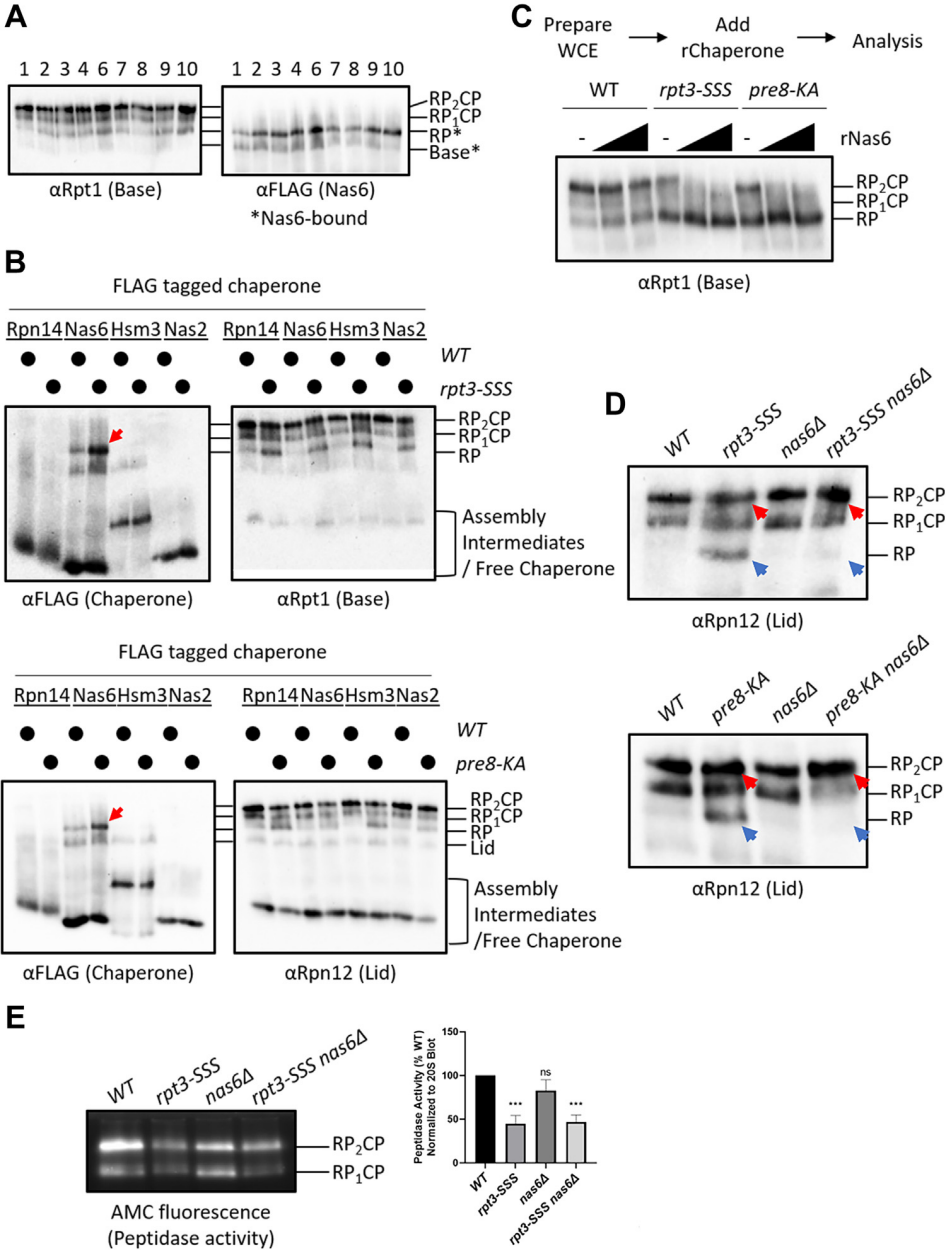
**RP assembly chaperone Nas6 associates with free RP in mutants with weakened RP–CP interfaces.***A*, native PAGE-immunoblotting of extracts from the mutants shown in Figure 2*A*. Each strain also expresses 3xFLAG-tagged Nas6 from the chromosomal locus. *B*, native PAGE-immunoblotting of extracts from *rpt3-SSS* (top panels) and *pre8-KA* (bottom panels) cells expressing the indicated 3xFLAG-tagged chaperones. A *red arrow* indicates accumulation of Nas6-bound RP. *C*, *top*, experimental workflow for Nas6-induced dissociation experiment. *Bottom*, native PAGE-immunoblotting of the indicated extracts pretreated with increasing concentrations of recombinant Nas6 (rNas6; 0, 0.1, or 1 μM). *D*, deletion of *NAS6* rescues the RP-CP association defect in *rpt3-SSS* and *pre8-KA* cells. *Red* and *blue* arrowheads indicate the changes in RP_2_CP and free RP, respectively observed *via* native PAGE. *E*, *NAS6* deletion rescues the structural defect of *rpt3-SSS* proteasomes, but not the peptidase defect. Native PAGE-separated extracts of the indicated strains were either immunoblotted for the CP or subjected to in-gel peptidase assay as described above. Peptidase activity was quantified as described in Figure 2*D*. One-way ANOVA with Tukey’s test for multiple comparisons; error bars represent ± SD; ns, not significant; ∗∗∗*p* < 0.001; *N* = 4. CP, core particle; RP, regulatory particle.

The accumulation of free RP observed in Rpt3 tail–docking mutant cells was similar to a phenotype our group had previously observed in a conformational mutant of the RP, called *rpn5-s1mut*. The *rpn5-s1mut* mutation compromised the release of Nas6 from nascent proteasomes upon completion of proteasome biogenesis, resulting in RP-CP dissociation and accumulation of Nas6-bound RP (16). This dissociation of RP from CP was thought to be due to a conformational defect that altered the RP-CP interface. We thus investigated whether Nas6 also accumulated on RP in the Rpt3 tail–docking mutant cells. Indeed, by native PAGE analysis, we observed a Nas6-containing band with a migration identical to the RP that was enriched in essentially all of the Rpt3 tail–docking mutant cells (Fig. 3*A*, lanes 3–10 *versus* lane 1), as well as in *pre8-KA* cells (Fig. 3*A*, lane 2). For further experiments, we chose the *rpt3-SSS* and *pre8-KA* cells as representative mutants with disrupted Rpt3 tail docking.

We next investigated whether other RP chaperones accumulated on RP in *rpt3-SSS* and *pre8-KA* cells using yeast strains expressing 3xFLAG-tagged versions of the four dedicated base assembly chaperones, Rpn14, Nas6, Nas2, and Hsm3. We analyzed extracts of these strains by native PAGE separation and immunoblotting for the chaperones to visualize the complexes with which they were associated. Notably, only Nas6 accumulated on free RP in these mutants (Fig. 3*B*, red arrows). Importantly, this was not due to an increase in total Nas6 expression (Fig. S2*A*).

In the case of the previously characterized *rpn5-s1mut*, Nas6 primarily acted prior to RP-CP association to prevent their stable interaction (16). This was demonstrated by adding recombinant Nas6 (rNas6) to purified *nas6Δ* RP prior to or after incubation with the CP. In this experiment, Nas6 could destabilize the RP-CP interaction only if added prior to RP-CP association. Thus, in the case of the Rpt3-docking mutants, if Nas6 exerts this function prior to docking of the RP onto the CP, then the addition of rNas6 to fully assembled, mature proteasomes will have no effect. In contrast, if Nas6 can actively dissociate mature proteasomes with defective Rpt3 tail docking, then the addition of rNas6 would be predicted to dissociate the RP from the CP. To distinguish between these possibilities, we added rNas6 to the extracts of WT, *rpt3-SSS*, or *pre8-KA* cells containing fully assembled, mature proteasomes and after incubation, visualized proteasome integrity by native PAGE-immunoblotting as above (Fig. 3*C*).

In the absence of rNas6 addition, an anti-Rpt1 (base subunit) blot revealed primarily RP_2_CP and RP_1_CP in WT extracts, with little free RP or base evident (Fig. 3*C*). Even in the presence of 10 μM rNas6, no appreciable loss of 26S proteasomes or accumulation of RP and CP was observed (Fig. S2*B*). In contrast, a concentration-dependent loss of RP-capped proteasomes (RP_2_CP and RP_1_CP) was triggered by rNas6 addition in the extracts of both *rpt3-SSS* and *pre8-KA* cells (Fig. 3*C*). Similar results were observed for the *rpt3-ext, rpt3-extHbYX, and rpt3-Δ1* mutants, whereas rNas6 was unable to destabilize fully formed proteasomes present in extracts of the *rpt3-ΔK416* mutant (Fig. S2*B*), in full agreement with the results above (Figs. 2, *B* and *D* and 3*A*).

To assess the specificity of this function for Nas6, we tested the impacts of adding the other two chaperones that can stably associate with the RP: Rpn14 and Hsm3. In contrast to what we observed for rNas6, addition of recombinant Rpn14 had no obvious effect on the integrity of WT or mutant proteasomes under any conditions tested (Fig. S2*C*). Intriguingly, recombinant Hsm3 appeared to promote dissociation of even WT proteasomes (Fig. S2*D*). However, Hsm3 did not accumulate on RP *in vivo* (Fig. 3*B*), suggesting this effect of Hsm3 is unlikely to be physiologically relevant. Together, these results indicate that Nas6 has a unique destabilizing role specifically for proteasomes in which Rpt3 tail docking is compromised. However, whereas Nas6 appears to primarily act prior to RP-CP association in the case of the previously reported *rpn5-s1mut*, Nas6 appears to be able to act on mature proteasomes in the case of these Rpt3 tail mutants.

In the Rpt3 tail–docking mutant cells, Nas6 may either passively accumulate on the free RP (Fig. 3*A*) or it may instead actively destabilize the RP-CP interface. If Nas6 actively destabilizes the RP-CP interface, then the deletion of *NAS6* would be predicted to rescue (or at least partially suppress) the assembly defect of these mutant proteasomes. To test this possibility, we analyzed the integrity of proteasomes in the extracts of *rpt3-SSS* or *pre8-KA* mutant cells that were either WT or null for *NAS6*. Notably, deletion of *NAS6* completely rescued the structural defect in both the *rpt3-SSS* and *pre8-KA* extracts (Fig. 3*D*), evident by loss of the accumulated RP (Fig. 3*D*, blue arrows) and a corresponding increase in doubly capped proteasomes (Fig. 3*D*, red arrows). However, deletion of *NAS6* was unable to rescue the peptidase defect observed in the *rpt3-SSS* mutant, indicating that the peptidase defect is independent of Nas6 (Fig. 3*E*).

We considered that Nas6 may associate with RP-CP to form a ternary complex that breaks down into Nas6-RP and CP only upon native gel electrophoresis. If so, then Nas6 would be anticipated to coimmunoprecipitate the CP in Rpt3 tail mutants. However, we detected no CP copurifying with Nas6 (Fig. S2*E*), arguing against this possibility. In summary, these observations suggest that Nas6 actively destabilizes the RP-CP interface, rather than passively accumulating on free RP or on RP-CP complexes that spontaneously dissociate to yield RP and CP.

### Nas6 preferentially destabilizes proteasomes with an RP-CP interface defect

**Figure 4:**
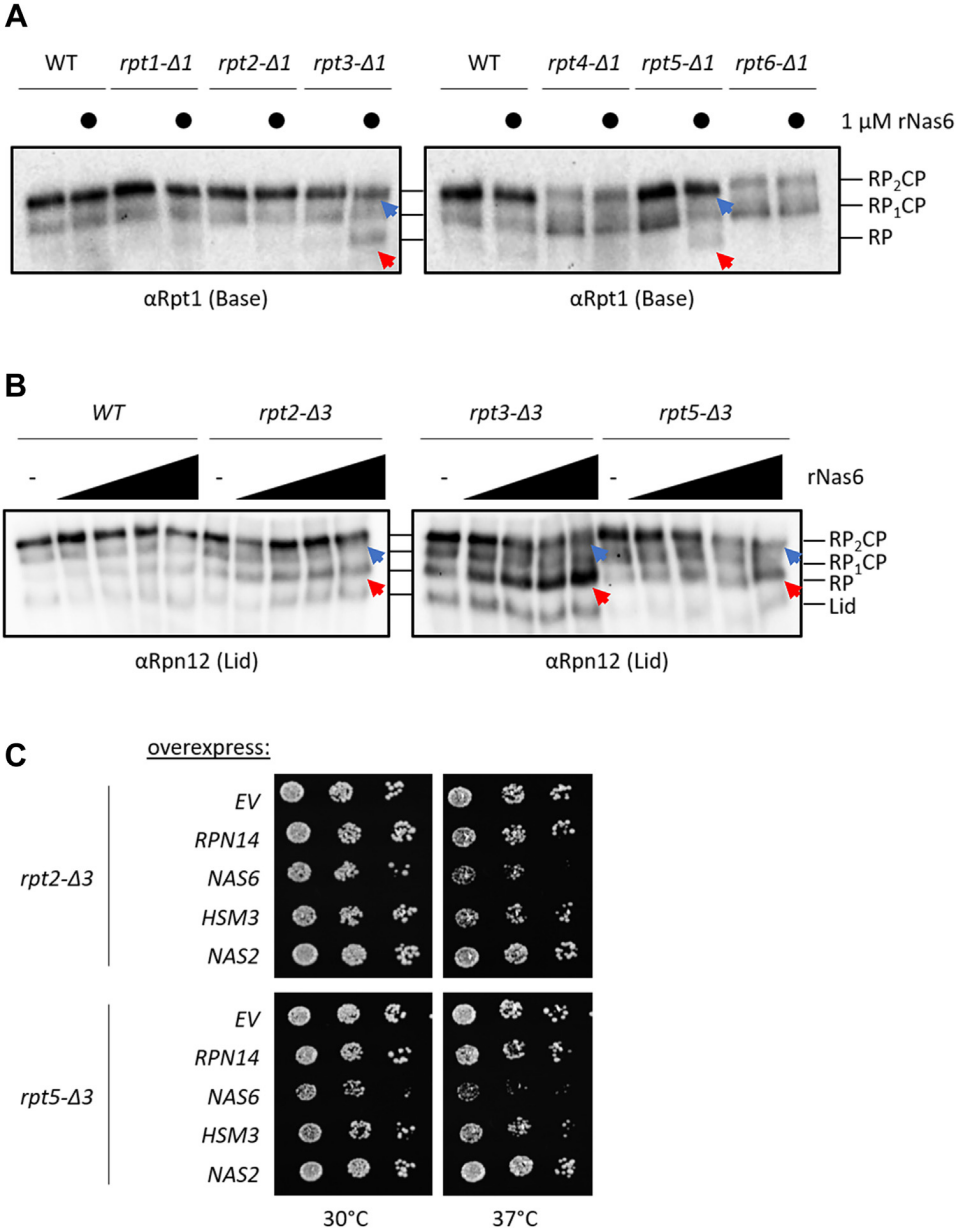
**Nas6 preferentially destabilizes proteasomes with an RP-CP defect.***A*, native PAGE-immunoblotting of the extracts of the indicated strains treated with or without recombinant Nas6 as shown in Figure 3*C*. *Blue* and *red arrows* indicate the loss of doubly capped proteasomes and accumulation of free RP, respectively. *B*, extracts of the indicated strains were analyzed and annotated as in (*A*). *C*, the strains shown were transformed with plasmids encoding the indicated proteins. The transformants were then spotted as serial dilutions onto selective media and incubated for 2 days at the temperatures shown before imaging. CP, core particle; RP, regulatory particle.

Given that Nas6 binds directly to the Rpt3 C-terminal helical domain immediately upstream of the Rpt3 C-terminal tail as part of its assembly function, we considered the possibility that this destabilizing role of Nas6 was a peculiarity specific to Rpt3 mutants. To test this, we added rNas6 to the extracts of cells in which the most C-terminal amino acid of each ATPase subunit was systematically deleted (*rptX-Δ1*) (10, 40). In addition to destabilizing proteasomes in the *rpt3-Δ1* mutant, rNas6 also destabilized *rpt5-Δ1* proteasomes (Fig. 4*A*, blue and red arrows). Like Rpt3, Rpt5 also contains a conserved HbYX motif that is constitutively docked into the surface of the CP (11, 12, 41); however, it is located nearly opposite Rpt3 in the ATPase ring, too far away to make any direct contact with Nas6.

Of note, Rpt2 also contains a conserved HbYX motif, but no disruption of RP-CP interaction was observed when rNas6 was added to *rpt2-Δ1* extracts. As the highly conserved HbYX tyrosine also makes important contacts in the α pocket (10, 42, 43), we hypothesized that the *rpt2-Δ1* mutant may not sufficiently weaken the RP-CP interface and that deleting the entire HbYX motif (*rpt2-Δ3*) may yield a similar phenotype upon addition of rNas6. Indeed, rNas6 also destabilized *rpt2-Δ3* proteasomes, albeit to a lesser extent than for Rpt3 mutants or the *rpt5-Δ1* (Fig. 4*A*) and *rpt5-Δ3* mutants (Fig. 4*B*, blue and red arrows). The relative destabilizing effect of Nas6 may depend upon the magnitude of the contributions of each HbYX motif to the affinity between the RP and CP. Consistent with these observations, overexpression of *NAS6* in the Rpt2 and Rpt5 HbYX truncation mutants led to impaired growth at elevated temperatures *in vivo* (Fig. 4*C*).

We also tested the other RP-associated chaperones against our panel of Rpt tail truncation mutants to establish whether any of the mutants were susceptible to destabilization by Rpn14 or Hsm3. We again found that adding rRpn14 (Fig. S3*A*) had no effect on any of the mutant Rpt tail truncation proteasomes under any conditions tested and that rHsm3 nonselectively destabilized all proteasomes, including WT (Fig. S3*B*). Additionally, we observed that overexpression of *NAS6* led to sick phenotypes at elevated temperatures in the Rpt3 and Rpt5 tail truncation mutants (Fig. S3*C*). In summary, these data indicate that Nas6 selectively disrupts the stability of mature proteasomes containing defects at the RP-CP interface.

### Nas6 has a specific and independent role in destabilizing proteasomes with a defective RP-CP interface

**Figure 5:**
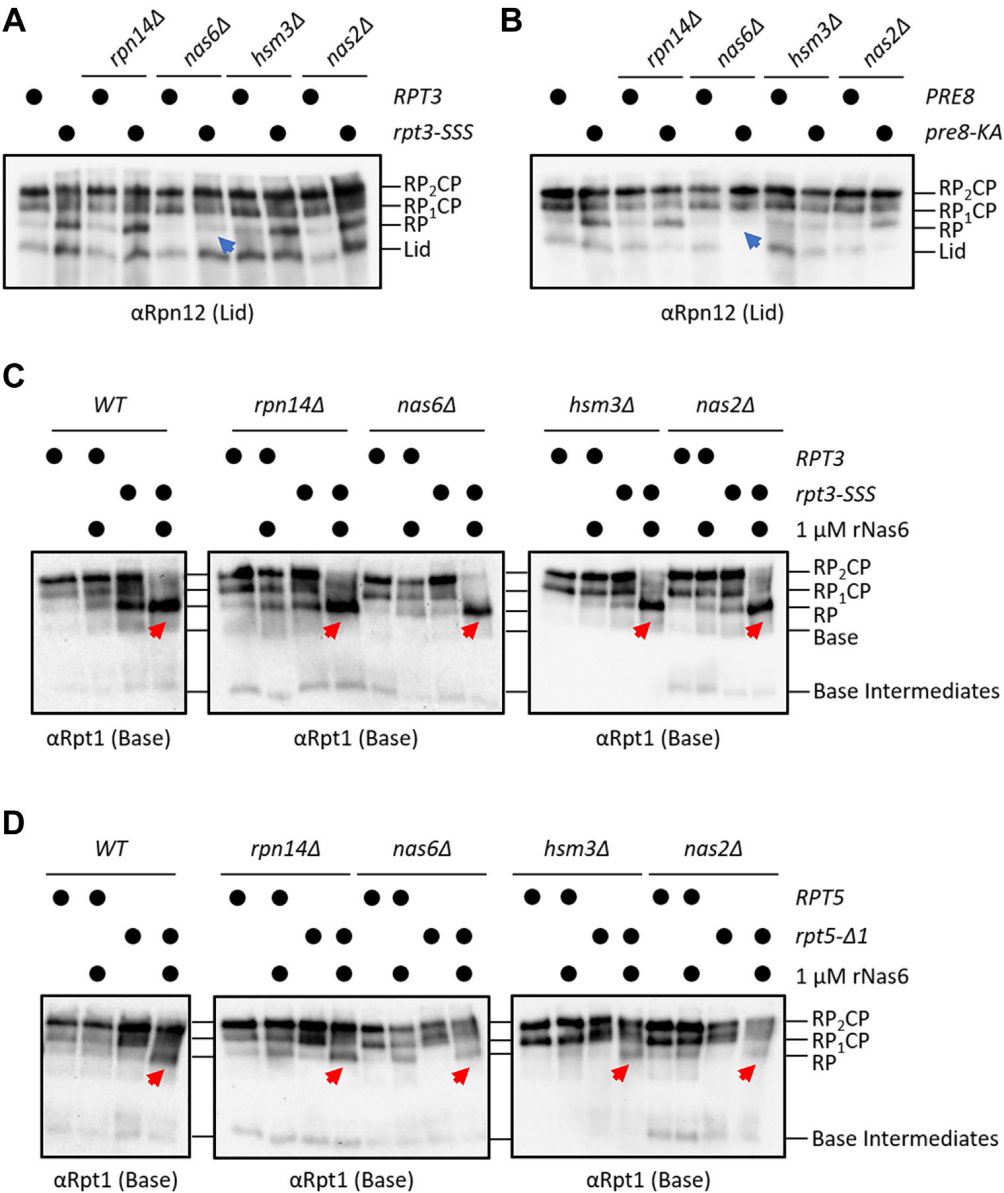
**Nas6 has a specific and independent role in destabilizing proteasomes with a defective RP-CP interface.***A* and *B*, native PAGE-immunoblotting of the indicated strains. A *blue arrowhead* indicates the rescue of RP accumulation upon deletion of *NAS6*. *C* and *D*, Nas6 can destabilize proteasomes in the absence of other RP chaperones. The indicated cell extracts were treated with rNas6 before native PAGE-immunoblotting. *Red arrows* indicate the accumulation of free RP. CP, core particle; RP, regulatory particle.

Genetic studies have strongly suggested that chaperones work cooperatively during the assembly process to promote proper RP biogenesis (17, 44). Whether Nas6 similarly cooperates with the other dedicated RP chaperones to fulfill this postassembly function remains unclear. To address this possibility, we tested whether deletion of each dedicated chaperone could ablate the accumulation of RP in *rpt3-SSS* and *pre8-KA* mutants as we had done for *NAS6* (Fig. 3*D*). However, we observed that only deletion of *NAS6*, and none of the other RP chaperones, significantly reduced the accumulation of RP in these mutants (Fig. 5, *A* and *B*, blue arrows). In further agreement, we found that Nas6 could destabilize proteasomes when each of the other three chaperones had been individually deleted. We added rNas6 to *rpt3-SSS* (Fig. 5*C*) or *rpt5-Δ1* (Fig. 5*D*) extracts in which *RPN14, NAS6, NAS2*, or *HSM3* had been deleted. Addition of rNas6 caused dissociation of mature proteasomes in the extracts of *rpt3-SSS* cells lacking each chaperone with the same apparent efficiency as in extracts with all chaperones present (Fig. 5*C*). The same was true when these experiments were repeated in *rpt5-Δ1* extracts systematically lacking each chaperone (Fig. 5*D*). Together, these data suggest that Nas6 exerts this destabilizing function independently of the other RP chaperones.

### The ability of Nas6 to dissociate RP and CP correlates with the RP-CP affinity

We envisioned an “affinity threshold” model for how Nas6 may selectively destabilize 26S proteasomes with defective RP-CP interfaces, yet be released from mature, functional (*e.g.*, WT) proteasomes. This is based on previous molecular modeling that demonstrated steric clash between Nas6 and the CP when Nas6 is modeled onto mature 26S proteasomes (15, 16). In this model, a ternary Nas6–RP-CP complex is formed either by transient Nas6 binding to mature proteasomes or upon docking of Nas6-RP onto CP during normal proteasome biogenesis. The eviction of Nas6 or CP would then be triggered by their steric clash with one another, with the species having the weaker affinity for the RP being released.

**Figure 6:**
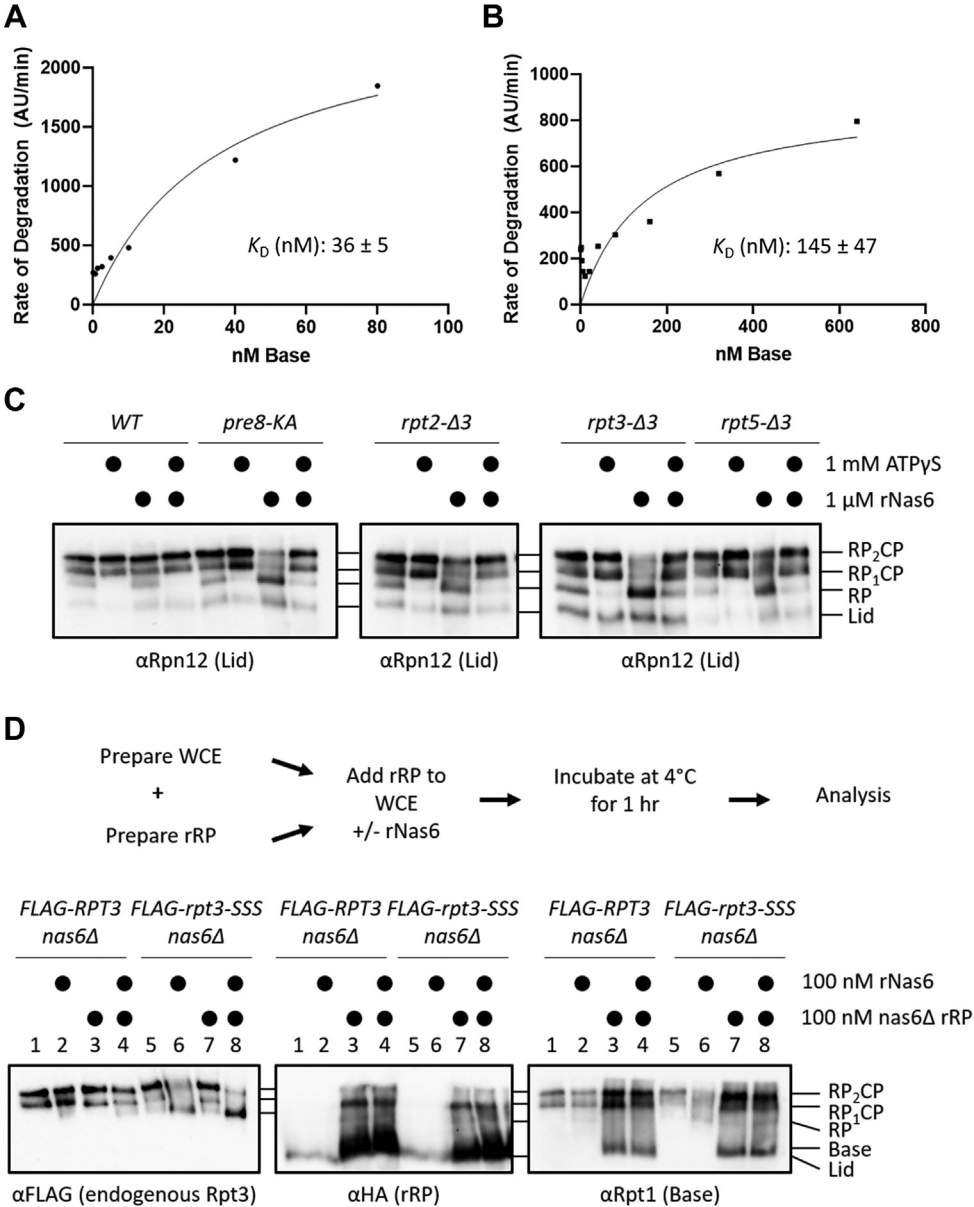
**The ability of Nas6 to dissociate RP and CP correlates with the RP-CP affinity.***A* and *B*, Michaelis–Menten analyses of base-CP interaction using WT (*A*) or *rpt3-Δ3* (*B*) base purified from *nas6Δ* cells. Note the different scales on the x-axes. The *K*_D_ for each is shown with the SD (*N* = 3). *C*, ATPγS prevents Nas6-dependent destabilization of proteasomes. Extracts of the indicated strains were prepared in the presence of ATP or ATPγS as indicated and then treated or not with rNas6 before separation by native PAGE and immunoblotting. *D*, *top*, experimental design for the measurement of recombinant RP (rRP) exchange. *Bottom*, native PAGE-immunoblots of the indicated strains incubated with increasing concentrations of rRP. CP, core particle; RP, regulatory particle.

At present, no quantitative assays for Nas6–RP interaction are available to permit direct comparison of the Nas6-RP affinity to the RP-CP affinity. However, the impact of modulating the RP-CP affinity of Nas6-dependent proteasome dissociation can be tested. As a first step, we reasoned that if Nas6 discriminated defective RP-CP interfaces based on a weakened interaction, then truncation of the Rpt3 tail as in the *rpt3-Δ3* mutant would weaken the affinity for the CP. To test this, we purified the base subcomplex from WT or *rpt3-Δ3* cells in which *NAS6* had been deleted (to avoid any interference from Nas6 on base–CP interaction). We then titrated increasing concentrations of *nas6Δ* or *nas6Δ rpt3-Δ3* base against a constant concentration of CP and measured the rate of degradation of a fluorogenic substrate, suc-LLVY-7-amino-4-methylcoumarin, as a surrogate readout for base–CP interaction. Fitting of initial rates to the Michaelis–Menten equation yielded a *K*_D_ of 36 ± 5 nM for *nas6Δ* base (Fig. 6*A*), which is similar to previous measurements for WT base presumably containing Nas6 (19, 39). As expected, *nas6Δ rpt3-Δ3* base displayed a substantially lower affinity for the CP (145 ± 47 nM; Fig. 6*B*). This represents an ∼4-fold poorer affinity *versus nas6Δ* base, consistent with the affinity threshold model, and indicating a substantial contribution of the Rpt3 tail to RP-CP affinity.

As a second test of this model, allosteric reinforcement of the RP-CP interface would be predicted to reduce the ability of Nas6 to promote the dissociation of defective proteasomes into RP-CP. The poorly hydrolyzed ATP analog ATPγS has previously been shown to strengthen the RP-CP interface (45, 46), presumably in part by driving insertion of the Rpt1 and Rpt6 C-termini into the surface of the CP (although it should be noted that other conformational changes may also contribute to this). We thus treated *pre8-KA, rpt2-Δ3, rpt3-Δ3*, and *rpt5*-Δ3 extracts with ATPγS prior to the addition of rNas6 and assayed the integrity of 26S proteasomes by native PAGE-immunoblotting (Fig. 6*C*). As we observed above, addition of rNas6 to extracts prepared in the presence of ATP selectively promoted the dissociation of proteasomes with defective RP-CP interfaces while sparing WT proteasomes. In contrast, preincubation of the same extracts with ATPγS prior to addition of rNas6 near-completely stabilized 26S proteasomes in all four of the mutants (Fig. 6*C*). Although these data cannot completely rule out other possibilities (*e.g.*, ATPγS alters the dynamics or conformation of RP-CP, or may alter the affinity of Nas6 for defective RP), the observations are together consistent with an affinity threshold model for Nas6-dependent destabilization of defective proteasomes.

### Nas6 does not alter the observed efficiency of WT RP-CP association in rpt3-SSS extracts

Finally, we sought to test whether Nas6 was functioning in part by accelerating the formation of new proteasomes when functional (*e.g.*, WT) base or RP was available, as Nas6 has been previously reported to regulate interaction between the base and CP (15). If so, then functional RP should be more efficiently incorporated into 26S proteasomes in the presence of rNas6 in *nas6Δ rpt3-SSS* extracts. To test this, we first mixed recombinant lid produced in *Escherichia coli* and base purified from *nas6Δ* yeast to permit the reconstitution of WT RP (rRP). We then added this mixture to the extracts of *RPT3 nas6Δ* or *rpt3-SSS nas6Δ* cells with or without added rNas6. To distinguish the endogenous RP from rRP, the extracts expressed *RPT3* or *rpt3-SSS* as N-terminal FLAG fusions from the native chromosomal locus (Fig. 6*D*, FLAG blot), and the rRP contained an HA tag on lid subunit Rpn7 (Fig. 6*D*, HA blot). As observed previously, addition of rNas6 displaced the endogenous mutant *FLAG-rpt3-SSS* RP, evident as a loss of FLAG signal in RP_2_CP and an accumulation of FLAG-containing RP complexes (Fig. 6*D*, FLAG blot, last four lanes). In contrast, addition of rNas6 to *FLAG-RPT3* extracts had no appreciable impact on FLAG signal in RP_2_CP or free RP (Fig. 6*D*, FLAG blot, first four lanes), consistent with our previous observations.

When *nas6Δ* rRP was added to *FLAG-RPT3* extracts, there was a modest accumulation of HA signal migrating similarly to RP_2_CP and RP_1_CP, indicating that rRP could associate with free CP present in the extracts (Fig. 6*D*, HA blot). This observation was supported by an enhancement of RP_2_CP and RP_1_CP levels in an Rpt1 (base subunit) blot of the same samples (Fig. 6*D*, Rpt1 blot), indicating that both the lid and base components of rRP were incorporated into new proteasomes. A similar increase in RP_2_CP and RP_1_CP levels was observed when *nas6Δ* rRP was added to *FLAG-rpt3-SSS* extracts. Additionally, we saw a resultant increase in peptidase activity of proteasomes formed from rRP for both *FLAG-RPT3* and *FLAG-rpt3-SSS* extracts (Fig. S4*A*, lanes 3, 4, 7, and 8). As no detectable peptidase activity was present in our purified lid or *nas6Δ* base complexes (Fig. S4*B*), and because proteasomes containing Rpt3 with defective tails had greatly reduced peptidase activity (Fig. 1*B*), this indicated that the RP assembling into 26S proteasomes under our assay conditions contained the exogenous base with a native Rpt3 C-terminus.

Although 26S proteasomes were formed efficiently from rRP in *rpt3-SSS* extracts, coprovision of rNas6 with rRP did not appreciably enhance the incorporation of rRP into 26S proteasomes (Fig. 6*D*, lanes 7 and 8 of HA and Rpt1 blots), indicating that the primary role of Nas6, at least under our assay conditions, is to dissociate defective proteasomes rather than to meaningfully enhance the assembly of new ones from functional RP and CP. In sum, our data support a model in which Nas6 serves a key postassembly quality control role by preventing the accumulation of proteasomes with defective meshing of the RP with the CP.

## Discussion

Here, we demonstrate that a previously described ability of Nas6 to destabilize defective proteasomes is substantially broader than initially appreciated and provide new insights into the mechanism(s) by which Nas6 discriminates them from functional proteasomes. We also highlight additional features of the Rpt3 C-terminus that are important for efficient docking into its cognate CP α pocket. A particularly unexpected observation was that Nas6 can destabilize mature proteasomes with a defective RP-CP interface after they have completed assembly, which has to our knowledge not been previously observed. We demonstrate that this postassembly function is unique to Nas6 and independent of other RP-associated chaperones. In light of these findings, we envision that Nas6 may fulfill a postassembly quality control function, scanning for and dissociating 26S proteasomes with defects at the RP-CP interface so that the functional subcomplexes harbored within them can be recycled into fully functional mature proteasomes.

We found that many alterations to the Rpt3 C-terminal tail, even outside of the conserved HbYX motif, caused an RP-CP defect that is characterized by the accumulation of Nas6-bound RP. The absence of other assembly chaperones on this accumulated RP immediately distinguished it from the Rpn14, Hsm3, and Nas6-bound RP generally agreed upon to be an assembly intermediate. Most studies examining the docking of Rpt subunit C-termini into the CP have focused on the highly conserved HbYX motifs present in Rpt2, Rpt3, and Rpt5. In contrast, the impact of HbYX motif spacing from the globular body of the ATPase subunit as well as how the sequence of amino acids immediately upstream of the HbYX motif impact RP–CP interaction have remained largely uninvestigated. Here, we found that alterations to the sequence upstream of the HbYX motif or to the spacing between the globular Rpt3 body and the HbYX motif caused defects of similar magnitude to deletion of the HbYX motif entirely. We speculate that many of these observations derive from disruption of the coiling of the Rpt3 tail on top of the α1-α2 pocket that is observed in available high-resolution structures. Future studies will be necessary to dissect the contributions of the contacts these upstream amino acids make with the CP to tail insertion and gating of the CP. We note that similar unstructured regions immediately preceding the HbYX motifs of Rpt2 and Rpt5 also appear to coil atop the CP in available structures of the 26S proteasome (11) and may similarly contribute to the affinity of the RP for the CP or for proper positioning of the HbYX motifs for docking.

**Figure 7:**
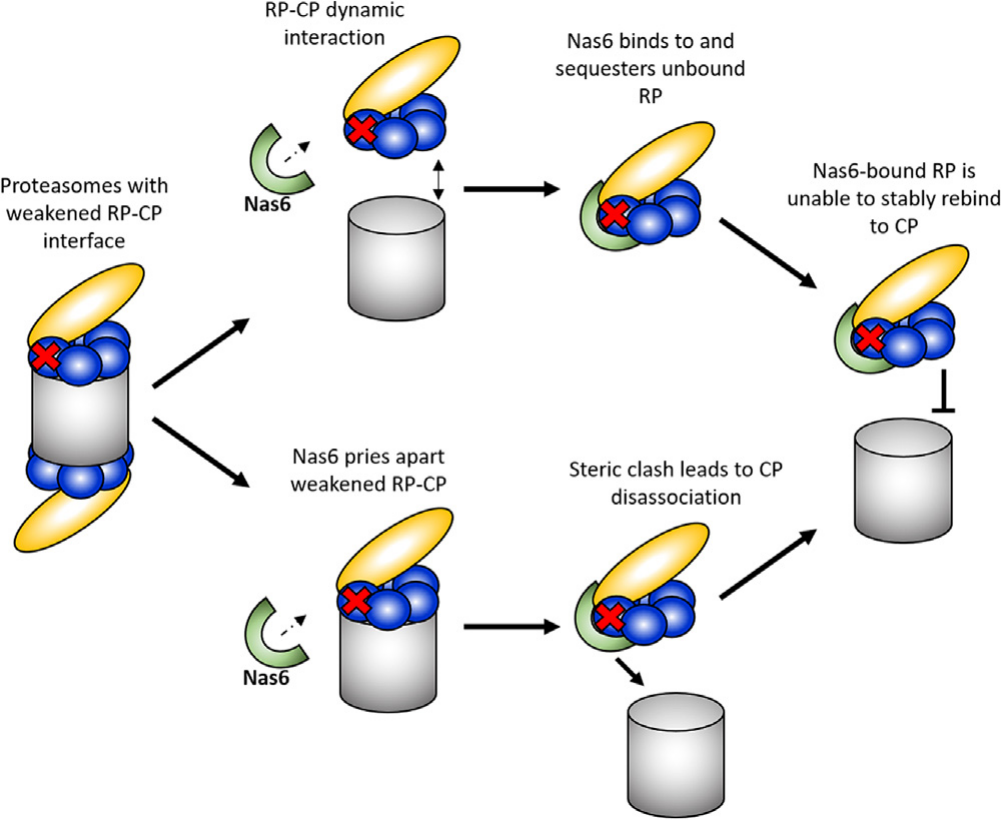
**Model for Nas6-dependent dissociation of proteasomes.** Nas6 may function either by sequestering RPs as they transiently dissociate from proteasomes with defective RP-CP interfaces (top path) and/or by forming a ternary complex with proteasomes, triggering dissociation of RP and CP and preventing rebinding (*bottom* path). See text for additional discussion. CP, core particle; RP, regulatory particle.

It is not yet clear whether Nas6 promotes dissociation of proteasomes with defective RP-CP interfaces by opportunistically binding and sequestering the RP during transient dissociation from the CP (Fig. 7, top pathway) or if instead it transiently binds to mature proteasomes harboring RP-CP interface defects and physically drives them apart (Fig. 7, bottom pathway). Molecular modeling indicates that Nas6 likely cannot stably occupy the RP simultaneously with the CP in the context of mature proteasomes (15, 16). Given that the interaction between the RP and CP is weakened in the *rpt3-Δ3* mutant tested here (Fig. 6*B*), and likely also in other RP-CP interface mutants, either or both mechanisms are possible. We note that our *K*_D_ estimate of ∼145 nM for *rpt3-Δ3* base–CP interaction is substantially weaker than for RP with an intact Rpt3 tail but much lower than the intracellular concentration of mature proteasomes (∼1 μM (47, 48, 49)). Thus, this mutation alone is unlikely to compromise RP–CP interaction to the extent observed here (Fig. 2*B*); the finding that deletion of Nas6 fully rescues the RP–CP interaction defect (Fig. 3*D*) also supports this supposition. Further, it has been previously noted that disruptions to the Rpt3 C-terminus can seemingly permit docking of Nas6 onto mature 26S proteasomes (18). For this reason, we favor a model where Nas6 actively promotes the dissociation of RP from CP. We posit that failed docking of particular Rpt C-termini can cause some “breathing” between the RP and CP that may permit Nas6 to bind to mature proteasomes; docking of the Rpt1 and Rpt6 C-termini in response to substrate engagement or nucleotide binding (7, 9, 16, 50) may then tighten the RP-CP interface, creating steric conflict that triggers dissociation of the less-tightly associated CP from the Nas6–RP complex. Quantitatively measuring the affinity and association/dissociation kinetics of Nas6 for the RP will help to establish the feasibility of this model.

It remains to be determined how Nas6 selectively destabilizes proteasomes with defective RP-CP interfaces. Nas6 is not known to make stable contact with the CP, but the accumulation of Nas6 on WT RP in cells harboring the *pre8-KA* suggests that Nas6 can destabilize proteasomes, harboring imperfections on either side of the RP-CP interface. Although our study focused primarily on defects arising from compromised docking of the conserved HbYX motifs into their cognate pockets on the surface of the CP, previous studies from our group and the Park group have demonstrated that Nas6 also destabilizes proteasomes with conformational defects arising from the compromise of contacts between the lid and base subcomplexes within the RP (15, 16). Although these contacts are distal to the RP-CP interface, they drive conformational switching of the proteasome for substrate degradation, and their disruption likely alters the interactions between the RP and CP. Molecular modeling of Nas6 onto mature 26S proteasomes (16, 17) indicates that it would reside at the RP-CP interface, poised to sense the meshing of these particles with one another.

We propose that Nas6 may fulfill a quality control function distinct from other known proteasome quality control pathways. Although Ecm29-dependent inactivation of defective proteasomes (29, 30) and autophagy of defective proteasomes (14, 31, 33) have been described, dissociation of defective proteasomes into subcomplexes would provide a key advantage not afforded by these mechanisms. Namely, the ability to partially dissociate defective proteasomes would in principle permit the recycling of the functional portions into nascent mature proteasomes. Although Ecm29 has been reported to bind and inactivate 26S proteasomes harboring defects in the Rpt5 C-terminus (30) similar to those utilized herein, we have found that this Nas6-dependent pathway occurs even in *ecm29Δ* cells (unpublished observations), suggesting they function in parallel rather than in cooperation. It is additionally unclear whether additional factors, such as posttranslational modifications or other chaperones or proteins are necessary for Nas6 to destabilize proteasomes with RP-CP interface defects. Such posttranslational modifications may serve to either alter the conformation, conformational switching, or other elements of the RP-CP interface to promote recruitment of Nas6 or enhance its ability to dissociate such defective proteasomes. Future studies will be needed to establish the relative contributions, triggers, and potential cofactors for each of these potential quality control mechanisms.

## Experimental procedures

### Yeast strains and media

Yeast strains used in this study are listed in Table S1. All yeast strains were created using standard genetic approaches (51) except as described below. Cells were grown in YPD or synthetic dropout medium at 30 °C unless otherwise indicated. Some pore mutant strains congenic with the RTY1 genetic background were kindly provided by Philip Coffino (Rockefeller University). The presence of the indicated pore mutations and the absence of secondary mutations were confirmed by DNA sequencing of the full *RPT* coding sequence prior to use.

The *pre8-K63A* strain was created using CRISPR/Cas9-mediated gene editing according to standard protocols (52). The starting strain was an α2 WT strain in which the chromosomal *PRE8* (α2) locus was marked with a hygromycin selectable marker cassette to permit facile tracking of the *pre8-K63A* allele in subsequent genetic crosses. The presence of the K63A substitution was confirmed by DNA sequencing of the full *PRE8* coding sequence prior to use.

### Plasmids

Plasmids used in this study are shown in Table S2. Plasmids were constructed using standard molecular cloning approaches with TOP10 F’ (Thermo Fisher Scientific) as the host strain. Site-directed mutagenesis was conducted *via* Quikchange (Agilent). All coding sequences were verified by DNA sequencing prior to use. Complete plasmid sequences and construct details are available upon request.

### Growth assays

Yeast strains were grown at 30 °C overnight to saturation and then diluted in deionized water to an A_600_ of 0.1. Six-fold serial dilutions were then made in sterile deionized water. These dilutions were then plated on growth media before incubation at the indicated temperatures for 2 to 5 days.

### Nondenaturing PAGE

Cell extracts were prepared and separated by native PAGE essentially as described previously (7, 16). Cells were grown in 500 ml of YPD media at 30 °C to log phase (A_600_ ≈ 1.0–2.0). Cultures were centrifuged at 5000*g* for 5 min, 4 °C. Cell pellets were then washed with 25 ml of ice-cold deionized water and centrifuged again at 4122*g*, 4 °C for 2 min. The supernatant was removed and the cell pellets were snap-frozen in liquid nitrogen. Cell pellets were crushed using a mortar and pestle precooled in liquid nitrogen. Cell powder was then hydrated with one volume of extraction buffer (50 mM Tris–HCl, pH 7.5, 5 mM MgCl_2_, 10% glycerol, 1 mM ATP, 0.015% w/v xylene cyanol) and vortexing frequently for 10 min on ice. Insoluble material was pelleted at 21,000*g* for 10 min at 4 °C, and 60 μg (measured by BCA assay) of the supernatant was applied to a 4% nondenaturing polyacrylamide gel containing 0.5 mM ATP and separated at 100 V, 4 °C.

### Denaturing PAGE (SDS-PAGE)

Cell extracts were prepared *via* the same method as described for native PAGE. 5× SDS loading buffer was added to the supernatants, applied to 10% SDS polyacrylamide gels and separated at 200 V at room temperature.

### Immunoblot analyses

Polyacrylamide gels were transferred to PVDF membranes at 100 V for 1 hour at 4 °C. Membranes were then incubated in 5% milk with antibodies against the CP (Enzo Life Sciences cat# PW9355, 1:5000), Rpt1 (53) (1:5000), Nas6 (gift from Mark Hochstrasser, 1:5000), FLAG (Sigma, cat#F3165, 1:5000), Rpn12 (7) (1:5000), Rpt3 (Enzo Life Sciences, cat#PW8245, 1:4000), G6PD (Sigma, cat#A9521, 1:20,000), HA (Covance, HA.11, 1:1000), Rpn5 (from Dan Finley, 1:5000). Blots were imaged on a Bio-Rad ChemiDoc MP using horseradish peroxidase–conjugated secondary antibodies (GE Healthcare) and ECL reagent.

### In-gel peptidase assays

Nondenaturing polyacrylamide gels were incubated in peptidase assay overlay buffer (50 mM Tris–HCl, pH 7.5, 5 mM MgCl_2_, 10% glycerol, 1 mM ATP) containing 50 μM suc-LLVY-7-amino-4-methylcoumarin substrate for 30 min at 30 °C with gentle shaking. Where indicated, 0.04% SDS was added to the overlay buffer before incubation to destabilize the CP gate. Gels were then imaged using a Bio-Rad ChemiDoc MP with the preprogrammed excitation and emission settings for ethidium bromide.

### Quantification and statistical analysis

Gel band volume intensities were calculated using Image Lab software (Bio-Rad; https://www.bio-rad.com/en-us/product/image-lab-software?ID=KRE6P5E8Z). 7-Amino-4-methylcoumarin fluorescence intensities were normalized to CP blot band intensities with the same loading. Statistical analysis was performed with Graph Pad Prism 9.0 software (https://www.graphpad.com/scientific-software/prism/) using a one-way ANOVA with Tukey’s test for multiple comparisons. Statistical significance was considered *p* < 0.05.

### CP affinity assays

The indicated amounts of base were preincubated with 2 nM CP at 30 °C for 15 min to allow for assembly. After preincubation, the reporter substrate suc-LLVY-7-amino-4-methylcoumarin was added to a final concentration of 5 μM. Fluorescence liberation kinetics were measured using a Biotek Synergy H1MF multimode reader. The initial rate of degradation (in AU/min) was plotted *versus* the concentration of the base (nM), and a hyperbolic curve was then fit to the data using Graph Pad Prism 9.0 software. The *K*_D_ and *B*_max_ were interpreted from the curve fit.

### Coimmunoprecipitation experiments

The indicated yeast strains were inoculated and grown in 2 l of YPD media at 30 °C with shaking to log phase (between 1.0 and 2.0 A_600_), and cultures were harvested by centrifugation at 5000*g* for 10 min, 4 °C. Pellets were washed in 50 mM Tris–HCl, pH 7.5 and then centrifuged as above. The supernatants were removed and pellets were snap-frozen in liquid nitrogen. Cell pellets were crushed using a mortar and pestle precooled in liquid nitrogen. To lyse cells, 5 ml of cell powder was thawed in 10 ml of buffer A (50 mM Tris–HCl, pH 7.5, 150 mM NaCl, 10% glycerol, 5 mM MgCl_2_, 2 mM ATP) with added 0.05% NP-40, 20 mM N-ethylmaleimide, and 2 mM PMSF and incubated on ice with frequent vortexing for 10 min. Cell debris was pelleted by centrifuging at 30,000*g* for 20 min at 4 °C. Supernatant was collected in a fresh container and 100 μl of packed FLAG resin was added to each sample. Whole cell extract was incubated with FLAG resin for 2 h with rocking at 4 °C. After 2 h, FLAG resin was washed twice with buffer A. Bound proteins were eluted from resin by incubating FLAG resin with buffer A containing three resin volumes of 200 μg/ml of 3xFLAG peptide on rotator for 45 min at 4 °C. The resin was pelleted and the supernatant with the eluted proteins was then applied to an SDS PAGE gel for analysis.

### Purification of recombinant lid from *E. coli*

Lid subunits from plasmids pRT2226 (pCDF42b-MBP-3Cx-Rpn6 - Rpn9 - Rpn11 - Rpn5 - Rpn8) and pRT2214 (pET42b-Rpn3 – Sem1 – HA-Rpn7 – 6His-Rpn12) were cotransformed in bacterial strain LOBSTR (DE3) containing pRARE2. Transformants were grown in 6 l of TB and the appropriate antibiotics at 37 °C, 250 rpm shaking until A_600_ = 1.0, at which point the temperature was reduced to 16 °C and IPTG was added to 0.5 mM. After overnight induction, cultures were centrifuged at 8000*g* for 5 min, 4 °C, the supernatant was poured off, and cells were frozen at −80 °C until purification. The day of purification, cells were thawed in Lid Lysis Buffer (50 mM Hepes–NaOH, pH 7.5, 100 mM NaCl, 100 mM KCl, 5% glycerol, 5 mM β-ME, 1 mM PMSF) and lysed with an Avestin Emulsiflex C-5. Lysates were clarified *via* centrifugation at 30,000*g*, 4 °C for 20 min. The supernatant was incubated with amylose resin for 30 min at 4 °C. After two washes with Lid Buffer (50 mM Hepes–NaOH, pH 7.5, 100 mM NaCl, 100 mM KCl, 5% glycerol, 5 mM β-ME), the resin was poured into a disposable Bio-Rad Econo-column, washed with Lid Buffer, and eluted with Lid Buffer containing 20 mM maltose. The eluate was then applied to Ni-NTA resin and incubated for 30 min at 4 °C. After two washes with Lid Buffer containing 10 mM imidazole, the resin was poured into a disposable Bio-Rad Econo-column, washed with Lid Buffer containing 10 mM imidazole, and eluted with Lid Buffer containing 250 mM imidazole. Eluates were concentrated using 100,000 Da MWCO filters (Amicon), and then the MBP tag was cleaved overnight by incubating the concentrated eluate with a 1:20 (w/w) ratio of HRV-3C protease overnight at 4 °C. The tag and protease were removed by gel filtration on a Superose 6 10-30 column equilibrated in Lid Buffer. Pure fractions were pooled, concentrated as above, and snap-frozen as small aliquots in liquid nitrogen for storage at −80 °C.

### Purification of recombinant base from *E. coli*

Base subunits from plasmids pRT2316 (pACYCDuet-1-tRNAs : Rpn14 : Nas6 : Nas2 : Hsm3), pRT1097 (pETDuet-1-Rpn1 : Rpn2 : Rpn13), and pRT1246 (pCOLADuet-1-MBP-3Cx-Rpt1 : Rpt2 : 6His-Rpt3 : Rpt5 : Rpt6 : Rpt4) were cotransformed in bacterial strain BL21-STAR (DE3). Transformants were grown in 6 l of 2xYT and the appropriate antibiotics at 37 °C, 250 rpm shaking until A_600_ = 0.7, at which point the temperature was reduced to 30 °C and IPTG was added to 0.5 mM. Cultures were grown for five additional hours at 30 °C, and then the temperature was reduced to 16 °C. After overnight induction, cultures were centrifuged at 8000 *g* for 5 min, 4 °C, the supernatant was poured off, and cells were resuspended in rBase Lysis Buffer (50 mM Hepes–NaOH, pH 7.5, 100 mM NaCl, 100 mM KCl, 10 mM MgCl_2_, 10% glycerol, 20 mM imidazole, 0.1% NP-40, 1 mM PMSF, 2 mM ATP, 1 mg/ml lysozyme, and 1 U/ml benzonase) and frozen at −80 °C until purification. The day of purification, cells were thawed and lysed *via* sonication. Lysates were clarified *via* centrifugation at 30,000*g*, 4 °C for 20 min. The supernatant was incubated with Ni-NTA resin and incubated for 45 min at 4 °C. After two washes with rBase Buffer (50 mM Hepes–NaOH, pH 7.5, 100 mM NaCl, 100 mM KCl, 10 mM MgCl_2_, 10% glycerol, 0.5 mM ATP) containing 20 mM imidazole, the resin was poured into a disposable Bio-Rad Econo-column, washed with rBase Buffer containing 20 mM imidazole, and eluted with rBase Buffer containing 250 mM imidazole. The eluate was then applied to amylose resin in a disposable Bio-Rad Econo-column. The flow-through was collected and again flowed over the amylose resin twice more. The resin was then washed with rBase Buffer containing 1 mM DTT, and proteins were eluted with rBase Buffer containing 1 mM DTT and 20 mM maltose. Eluates were concentrated using 100,000 Da MWCO filters (Amicon), and then the MBP tag was cleaved by incubating the concentrated eluate with HRV-3C protease overnight at 4 °C as described for the lid. The reaction was further purified by gel filtration on a Superose 6 column equilibrated in rBase Buffer. Pure fractions were pooled, concentrated as above, and snap-frozen as small aliquots in liquid nitrogen for storage at −80 °C.

### Purification of recombinant base from yeast

Yeast base was purified from cells containing an *RPN2-link-3Cx-link-2xALFA:kanMX6* allele for affinity purification *via* ALFA nanobody (Nb) resin. The indicated yeast strains were grown in YPD media with 4% glucose at 30 °C, 250 rpm for 48 h. Yeast were harvested by centrifugation at 5000*g*, 25 °C for 5 min. The supernatant was discarded and the pellet resuspended in 1 l of ice-cold dH_2_O and then centrifuged as before. Cell pellets were then scooped into liquid nitrogen to snap-freeze and then crushed into powder *via* the SPEX Cryomill. Cell powder was then stored at −80 °C until purification. On the day of purification, cell powder was first thawed in an equal volume of yBase Buffer (50 mM Hepes–NaOH, pH 7.5, 50 mM NaCl, 50 mM KCl, 10 mM MgCl_2_, 5% glycerol, 0.5 mM ATP) supplemented with 0.05% NP-40 with gentle stirring until completely thawed and then incubated an additional 10 min. Lysates were then clarified *via* centrifugation at 30,000*g*, 4 °C for 20 min. The supernatant was poured through three thicknesses of cheesecloth to remove lipids and then incubated with ALFA nanobody resin for 90 min at 4 °C with rocking. After two washes with yBase Wash Buffer (50 mM Hepes–NaOH, pH 7.5, 500 mM NaCl, 10 mM MgCl_2_, 10% glycerol, 0.1% NP-40, 0.5 mM ATP), the resin was then incubated with yBase Buffer containing 5 mM DTT and 500 μg HRV-3C protease overnight at 4 °C with end-over-end nutation. The following day, the eluate was collected, and two additional washes with yBase Buffer were collected and combined with the eluate. The combined eluate and washes were concentrated using 100,000 Da MWCO filters (Amicon) and further purified by gel filtration on a Superose 6 column equilibrated in yBase Buffer. Pure fractions were pooled, concentrated as above, and snap-frozen as small aliquots in liquid nitrogen for storage at −80 °C.

### Purification of ALFA Nb and conjugation to resin for purification

ALFA Nb was expressed as an N-terminal 14His fusion from plasmid pRT2591 in bacterial strain LOBSTR (DE3) cotransformed with pRARE2. Transformants were grown in 6 l of LB and the appropriate antibiotics at 37 °C, 250 rpm shaking until A_600_ = 1.0, at which point the temperature was reduced to 16 °C and IPTG was added to 0.2 mM. After overnight induction, cultures were centrifuged at 8000*g* for 5 min, 4 °C, the supernatant was poured off, and cells were frozen at −80 °C until purification. The day of purification, cells were thawed in Tris–NTA Buffer (50 mM Tris–HCl, pH 7.5, 500 mM NaCl, 0.2% Tween-20, 10% glycerol, 20 mM imidazole, 1 mM TCEP) supplemented with 1 mM PMSF and lysed with an Avestin Emulsiflex C-5. Lysates were clarified *via* centrifugation at 30,000*g*, 4 °C for 20 min. The supernatant was incubated with Ni-NTA resin for 30 min at 4 °C. After two washes with Tris–NTA Buffer, the resin was poured into a disposable Bio-Rad Econo-column, washed with Tris–NTA Buffer and eluted by incubating with the cleavage buffer (Tris–NTA Buffer with 70 mM imidazole, 3 mM TCEP, 250 nM *Brachypodium distachyon* NEDP1 (54)) for 2 h at 4 °C. The eluate was then collected, and the resin was washed twice more with Tris–NTA + 70 mM TCEP. These two washes were combined with the eluate and concentrated using a 3000 Da MWCO filter (Amicon) and further purified by gel filtration on a Sephacryl S-200 column equilibrated in Lid Buffer. Pure fractions were pooled and concentrated to approximately 5 mg/ml.

The purified ALFA Nb was then covalently coupled to SulfoLink Coupling Resin (Pierce #20401). All reagents were equilibrated to room temperature and the purified ALFA Nb was added to the resin at a ratio of 5 mg Nb: 1 ml packed resin. These were incubated at room temperature with end-over-end nutation for 45 min in the dark. The resin was then washed with Nb Coupling Buffer (50 mM Tris–HCl, pH 8.5, 150 mM NaCl, 5 mM EDTA, 0.5 mM TCEP), and then the reaction was quenched by adding Nb Coupling Buffer containing 50 mM DTT. The quenching reaction was incubated at room temperature for 45 min with end-over-end nutation. The resin was washed twice with Nb Coupling Buffer supplemented with 1 M NaCl and then washed with Nb Resin Storage Buffer (1× PBS in 20% ethanol). The ALFA Nb resin was then stored at 4 °C in Nb Resin Storage Buffer until use.

### Purification of recombinant assembly chaperones

Nas6 was expressed as a C-terminal 6His fusion from plasmid pRT37 in bacterial strain LOBSTR (DE3) cotransformed with pRARE2. Transformants were grown in 2 l of LB and the appropriate antibiotics at 37 °C, 250 rpm shaking until A_600_ = 0.6, at which point the temperature was reduced to 16 °C and IPTG was added to 0.5 mM. After overnight induction, cultures were centrifuged at 8000*g* for 5 min, 4 °C, the supernatant was poured off, and cells were frozen at −80 °C until purification. The day of purification, cells were thawed in NPI-10 (10 mM sodium phosphate, pH 8.0, 300 mM NaCl, 10 mM imidazole) and lysed with an Avestin Emulsiflex C-5. Lysates were clarified *via* centrifugation at 30,000*g*, 4 °C for 20 min. The supernatant was incubated with Ni-NTA resin for 30 min at 4 °C. After two washes with NPI-10, the resin was poured into a disposable Bio-Rad Econo-column, washed with NPI-20 (10 mM sodium phosphate, pH 8,0, 300 mM NaCl, 20 mM imidazole), and eluted with NPI-500 (10 mM sodium phosphate, pH 8.0, 100 mM NaCl, 500 mM imidazole). Eluates were concentrated using 10,000 Da MWCO filters (Amicon) and further purified by gel filtration on a Sephacryl S-200 column equilibrated in Lid Buffer (50 mM HEPES·OH, pH 7.5, 100 mM NaCl, 100 mM KCl, 5% glycerol). Pure fractions were pooled, concentrated as above, and snap-frozen as small aliquots in liquid nitrogen for storage at −80 °C.

Rpn14 was expressed as a C-terminal 6His fusion from plasmid pRT39 and purified exactly as for Nas6. Hsm3 was expressed with an N-terminal 12His-SUMO tag exactly as for Nas6 and Rpn14. After lysis and binding to the Ni-NTA resin, the resin was washed with one column volume of SUMO cleavage buffer (50 mM Tris·Cl, pH 7.5, 500 mM NaCl, 10% glycerol, 65 mM imidazole, 0.2% Tween-20, 2 mM TCEP) and incubated with cleavage buffer containing 250 nM *B. distachyon* SENP1 (54) for 2 h at 4 °C to cleave Hsm3 from the affinity tag. The flow-through was collected, concentrated, and further purified on a Sephacryl S-200 column equilibrated in Lid Buffer (50 mM HEPES·OH, pH 7.5, 100 mM NaCl, 100 mM KCl, 5% glycerol). Pure fractions were pooled, concentrated as above, and snap-frozen as small aliquots in liquid nitrogen for storage at −80 °C.

### Reconstituted RP assembly assays

Extracts from the indicated yeast strains were prepared as described for analysis for nondenaturing PAGE. Total protein concentration was calculated *via* BCA assay, and then all samples were diluted to 10 mg/ml in extraction buffer. Reconstituted RP was formed by incubating equimolar concentrations of recombinant base and recombinant lid together in extraction buffer for 15 min at 30 °C. Then, reconstituted RP was added to indicated extracts at indicated concentrations and incubated on ice for an hour, with frequent gentle mixing (Fig. 6*D*).

## Data availability

All data are contained in the article. Requests for yeast strains or plasmids should be sent to: robert.tomko@med.fsu.edu.

## Supporting information

This article contains supporting information (18, 55, 56, 57).

## Conflict of interest

The authors declare that they no conflicts of interest with the contents of this article.
