# Supplementary material for: Assembly chaperone Nas6 selectively destabilizes 26S proteasomes with defective regulatory particle-core particle interfaces
Source: J Biol Chem. 2023 Jan 10;299(2):102894. doi: 10.1016/j.jbc.2023.102894 (PMC9943895; doi:10.1016/j.jbc.2023.102894)
Supplement: Supplemental Tables S1–S2 and Figures S1–S4 [file mmc1.pdf]

Assembly chaperone Nas6 selectively destabilizes 26S proteasomes with defective regulatory particle-core particle interfaces

Jennifer L. Warnock, Gabriel W. Jobin, Sandhya Kumar, and Robert J. Tomko Jr.

Supplemental Tables

Supplemental Table S1 related to all figures: yeast strains used in this study.

Strain	Genotype	Source
**RTY1**	*MATa his3-Δ200 leu2-3,112 ura3-52 lys2-801 trp1-1 gal2*	Lab background
**RTY518**	*MATa his3-Δ200 leu2-3,112 ura3-52 lys2-801 trp1-1 gal2 rpt3+1:kanMX4*	This study
**RTY980**	*MATa his3-Δ200 leu2-3,112 ura3-52 lys2-801 trp1-1 gal2 rpn4Δ::kanMX4*	This study
**RTY1000**	*MATa his3-Δ200 leu2-3,112 ura3-52 lys2-801 trp1-1 gal2 nas2Δ::HIS3*	This study
**RTY1002**	*MATa his3-Δ200 leu2-3,112 ura3-52 lys2-801 trp1-1 gal2 hsm3Δ::kanMX4*	This study
**RTY1178**	*MATa his3-Δ200 leu2-3,112 ura3-52 lys2-801 trp1-1 gal2 rpt6-Δ1:natMX4*	Park et al. 2009[1]
**RTY1664**	*MATa his3-Δ200 leu2-3,112 ura3-52 lys2-801 trp1-1 gal2 rpt1-Δ1:kanMX6*	Park et al. 2009[1]
**RTY1665**	*MATa his3-Δ200 leu2-3,112 ura3-52 lys2-801 trp1-1 gal2 rpt2-Δ1:kanMX6*	Park et al. 2009[1]
**RTY1666**	*MATa his3-Δ200 leu2-3,112 ura3-52 lys2-801 trp1-1 gal2 rpt4-Δ1:natMX4*	Park et al. 2009[1]
**RTY1667**	*MATa his3-Δ200 leu2-3,112 ura3-52 lys2-801 trp1-1 gal2 rpt5-Δ1:natMX4*	Park et al. 2009[1]
**RTY1691**	*MATa his3-Δ200 leu2-3,112 ura3-52 lys2-801 trp1-1 gal2 rpt3-Δ1:kanMX6*	This study
**RTY1891**	*MATa his3-Δ200 leu2-3,112 ura3-52 lys2-801 trp1-1 gal2 rpn14Δ::kanMX4*	This study
**RTY1895**	*MATa his3-Δ200 leu2-3,112 ura3-52 lys2-801 trp1-1 gal2 nas6Δ::kanMX4*	This study
**RTY2154**	*MATa his3-Δ200 leu2-3,112 ura3-52 lys2-801 trp1-1 gal2 NAS6-6xGly-3xFLAG:kanMX6*	This study
**RTY2245**	*MATa his3-Δ200 leu2-3,112 ura3-52 lys2-801 trp1-1 gal2 HSM3-6xGly-3xFLAG:hphMX4*	This study
**RTY2522**	*MATa his3-Δ200 leu2-3,112 ura3-52 lys2-801 trp1-1 gal2 rpt3(Y246A):natMX4*	This study
**RTY2667**	*MATa his3-Δ200 leu2-3,112 ura3-52 lys2-801 trp1-1 gal2 rpt3(Y246A),ext:hphMX4*	This study
**RTY2853**	*MATa his3-Δ200 leu2-3,112 ura3-52 lys2-801 trp1-1 gal2 rpt3(Y246A),ext:hphMX4 rpn4Δ::kanMX6*	This study

**RTY3157**	*MATa his3-Δ200 leu2-3,112 ura3-52 lys2-801 trp1-1 gal2 RPN14-6xGly-3xFLAG:kanMX6*	This study
**RTY3159**	*MATa his3-Δ200 leu2-3,112 ura3-52 lys2-801 trp1-1 gal2 NAS2-6xGly-3xFLAG:kanMX6*	This study
**RTY3167**	*MATa his3-Δ200 leu2-3,112 ura3-52 lys2-801 trp1-1 gal2 NAS6-6xGly-3xFLAG:kanMX6 rpt3-YA,ext:hphMX4*	This study
**RTY3584**	*MATa his3-Δ200 leu2-3,112 ura3-52 lys2-801 trp1-1 gal2 rpt5-Δ1:natMX4 nas6Δ::kanMX4*	This study
**RTY3597**	*MATa his3-Δ200 leu2-3,112 ura3-52 lys2-801 trp1-1 gal2 pre8-K63A::hphMX4*	This study
**RTY3633**	*MATa his3-Δ200 leu2-3,112 ura3-52 lys2-801 trp1-1 gal2 rpt3-Δ1:kanMX6 NAS6-6xGly-3xFLAG:kanMX6*	This study
**RTY3635**	*MATa his3-Δ200 leu2-3,112 ura3-52 lys2-801 trp1-1 gal2 rpt3+1:kanMX6 NAS6-6xGly-3xFLAG:kanMX6*	This study
**RTY3687**	*MATa his3-Δ200 leu2-3,112 ura3-52 lys2-801 trp1-1 gal2 RPN2-link-3Cx-link-2xALFA:kanMX6 rpn10Δ::HIS3 nas6Δ::natMX4*	This study
**RTY3714**	*MATa his3-Δ200 leu2-3,112 ura3-52 lys2-801 trp1-1 gal2 rpt3-SSS:HIS3MX6*	This study
**RTY3715**	*MATa his3-Δ200 leu2-3,112 ura3-52 lys2-801 trp1-1 gal2 rpt3-Δ3:HIS3MX6*	This study
**RTY3718**	*MATa his3-Δ200 leu2-3,112 ura3-52 lys2-801 trp1-1 gal2 rpt3-ext:HIS3MX6*	This study
**RTY3732**	*MATa his3-Δ200 leu2-3,112 ura3-52 lys2-801 trp1-1 gal2 rpt3-SSS:HIS3MX6 NAS6-6xGly-3xFLAG:kanMX6*	This study
**RTY3734**	*MATα his3-Δ200 leu2-3,112 ura3-52 lys2-801 trp1-1 gal2 rpt3-Δ3:HIS3MX6 NAS6-6xGly-3xFLAG:kanMX6*	This study
**RTY3735**	*MATa his3-Δ200 leu2-3,112 ura3-52 lys2-801 trp1-1 gal2 rpt3-ext:HIS3MX6 NAS6-6xGly-3xFLAG:kanMX6*	This study
**RTY3737**	*MATa his3-Δ200 leu2-3,112 ura3-52 lys2-801 trp1-1 gal2 pre8-K63A:hphMX4 NAS6-6xGly-3xFLAG:kanMX6*	This study
**RTY3742**	*MATa his3-Δ200 leu2-3,112 ura3-52 lys2-801 trp1-1 gal2 pre8-K63A:hphMX4 rpn4Δ::hphMX4*	This study
**RTY3744**	*MATa his3-Δ200 leu2-3,112 ura3-52 lys2-801 trp1-1 gal2 rpt3-ext:HIS3MX6 rpn4Δ::hphMX4*	This study
**RTY3745**	*MATa his3-Δ200 leu2-3,112 ura3-52 lys2-801 trp1-1 gal2 rpt3-extHbYX:HIS3MX6*	This study
**RTY3746**	*MATa his3-Δ200 leu2-3,112 ura3-52 lys2-801 trp1-1 gal2 rpt3-SSS:HIS3MX6 nas6Δ::kanMX4*	This study
**RTY3752**	*MATa his3-Δ200 leu2-3,112 ura3-52 lys2-801 trp1-1 gal2 pre8-K63A:hphMX4 nas6Δ::kanMX4*	This study
**RTY3754**	*MATa his3-Δ200 leu2-3,112 ura3-52 lys2-801 trp1-1 gal2 rpt3-extHbYX:HIS3MX6 NAS6-6xGly-3xFLAG:kanMX6*	This study
**RTY3756**	*MATa his3-Δ200 leu2-3,112 ura3-52 lys2-801 trp1-1 gal2 rpt3-Δ1:kanMX6 rpn4Δ::hphMX4*	This study
**RTY3774**	*MATa his3-Δ200 leu2-3,112 ura3-52 lys2-801 trp1-1 gal2 rpt3-ΔK416:HIS3MX6*	This study

**RTY3780**	*MATa his3-Δ200 leu2-3,112 ura3-52 lys2-801 trp1-1 gal2 rpt3-5A:HIS3MX6*	This study
**RTY3782**	*MATa his3-Δ200 leu2-3,112 ura3-52 lys2-801 trp1-1 gal2 pre8-K63A:hphMX4 nas2Δ::HIS3MX6*	This study
**RTY3785**	*MATa his3-Δ200 leu2-3,112 ura3-52 lys2-801 trp1-1 gal2 rpt3-SSS:HIS3MX6 nas2Δ::HIS3MX6*	This study
**RTY3787**	*MATa his3-Δ200 leu2-3,112 ura3-52 lys2-801 trp1-1 gal2 pre8-K63A:hphMX4 hsm3Δ::kanMX4*	This study
**RTY3789**	*MATa his3-Δ200 leu2-3,112 ura3-52 lys2-801 trp1-1 gal2 rpt3-SSS:HIS3MX6 hsm3Δ::kanMX4*	This study
**RTY3792**	*MATα his3-Δ200 leu2-3,112 ura3-52 lys2-801 trp1-1 gal2 pre8-K63A:hphMX4 rpn14Δ::kanMX4*	This study
**RTY3797**	*MATa his3-Δ200 leu2-3,112 ura3-52 lys2-801 trp1-1 gal2 rpt3-SSS:HIS3MX6 rpn14Δ::kanMX4*	This study
**RTY3801**	*MATa his3-Δ200 leu2-3,112 ura3-52 lys2-801 trp1-1 gal2 rpt3-SSS:HIS3MX6 HSM3-6xGly-3xFLAG:hphMX4*	This study
**RTY3803**	*MATa his3-Δ200 leu2-3,112 ura3-52 lys2-801 trp1-1 gal2 rpt3-SSS:HIS3MX6 RPN14-6xGly-3xFLAG:kanMX6*	This study
**RTY3805**	*MATa his3-Δ200 leu2-3,112 ura3-52 lys2-801 trp1-1 gal2 rpt3-SSS:HIS3MX6 NAS2-6xGly-3xFLAG:kanMX6*	This study
**RTY3819**	*MATa his3-Δ200 leu2-3,112 ura3-52 lys2-801 trp1-1 gal2 pre8-K63A:hphMX4 RPN14-6xGly-3xFLAG:kanMX6*	This study
**RTY3821**	*MATa his3-Δ200 leu2-3,112 ura3-52 lys2-801 trp1-1 gal2 pre8-K63A:hphMX4 NAS2-6xGly-3xFLAG:kanMX6*	This study
**RTY3823**	*MATa his3-Δ200 leu2-3,112 ura3-52 lys2-801 trp1-1 gal2 pre8-K63A:hphMX4 HSM3-6xGly-3xFLAG:hphMX4*	This study
**RTY3838**	*MATa his3-Δ200 leu2-3,112 ura3-52 lys2-801 trp1-1 gal2 rpt3-5A:HIS3MX6 NAS6-6xGly-3xFLAG:kanMX6*	This study
**RTY3846**	*MATa his3-Δ200 leu2-3,112 ura3-52 lys2-801 trp1-1 gal2 rpt5-Δ1:natMX4 nas2Δ::HIS3*	This study
**RTY3848**	*MATa his3-Δ200 leu2-3,112 ura3-52 lys2-801 trp1-1 gal2 rpt5-Δ1:natMX4 hsm3Δ::kanMX4*	This study
**RTY3850**	*MATa his3-Δ200 leu2-3,112 ura3-52 lys2-801 trp1-1 gal2 rpt5-Δ1:natMX4 rpn14Δ::kanMX4*	This study
**RTY3895**	*MATa his3-Δ200 leu2-3,112 ura3-52 lys2-801 trp1-1 gal2 rpt2-Δ3:HIS3MX6*	This study
**RTY3897**	*MATa his3-Δ200 leu2-3,112 ura3-52 lys2-801 trp1-1 gal2 rpt5-Δ3:HIS3MX6*	This study
**RTY3905**	*MATa/α his3-Δ200/his3-Δ200 leu2-3,112/leu2-3,112 ura3-52/ura3-52 lys2-801/lys2-801 trp1-1/trp1-1 gal2/gal2 FLAG-TEVx-RPT3:HIS3MX6/FLAG-TEVx-RPT3:HIS3MX6*	This study
**RTY3906**	*MATa/α his3-Δ200/his3-Δ200 leu2-3,112/leu2-3,112 ura3-52/ura3-52 lys2-801/lys2-801 trp1-1/trp1-1 gal2/gal2 FLAG-TEVx-RPT3:HIS3MX6/FLAG-TEVx-RPT3:HIS3MX6 nas6Δ::kanMX4/nas6Δ::kanMX4*	This study

**RTY3985**	*MATa/α his3-Δ200/his3-Δ200 leu2-3,112/leu2-3,112 ura3-52/ura3-52 lys2-801/lys2-801 trp1-1/trp1-1 gal2/gal2 FLAG-TEVx-rpt3-SSS:HIS3MX6/FLAG-TEVx-rpt3-SSS:HIS3MX6*	This study
**RTY3986**	*MATa/α his3-Δ200/his3-Δ200 leu2-3,112/leu2-3,112 ura3-52/ura3-52 lys2-801/lys2-801 trp1-1/trp1-1 gal2/gal2 FLAG-TEVx-rpt3-SSS:HIS3MX6/FLAG-TEVx-rpt3-SSS:HIS3MX6 nas6Δ::kanMX4/nas6Δ::kanMX4*	This study
**RTY4017**	*MATa his3-Δ200 leu2-3,112 ura3-52 lys2-801 trp1-1 gal2 RPN2-link-3Cx-link-2xALFA:kanMX6 rpn10Δ::HIS3 nas6Δ::natMX4 rpt3-Δ3:HIS3MX6*	This study

Supplemental Table S2 related to all figures: plasmids used in this study.

Plasmid	Genotype	Source
**pRT7**	YCplac33-*RPT3*	This study
**pRT37**	pET42b-Nas6-6His	This study
**pRT39**	pET42b-Rpn14-6His	This study
**pRT54**	p414GPD	Mumberg et al. 1995[2]
**pRT128**	p426GPD	Mumberg et al. 1995[2]
**pRT131**	p414GPD-Rpn14	This study
**pRT170**	YCplac33	Gietz and Sugino 1988[3]
**pRT452**	p414GPD-Nas6	This study
**pRT1097**	pETDuet-1-Rpn1 : Rpn2 : Rpn13	Beckwith et al. 2013[4]
**pRT1246**	pCOLADuet-1-MBP-3Cx-Rpt1 : Rpt2 : 6His-Rpt3 : Rpt5 : Rpt5 : Rpt6 : Rpt4	This study
**pRT1929**	p426GPD-Nas6	This study
**pRT1951**	p426GPD-Rpn14	This study
**pRT1952**	p426GPD-Hsm3	This study
**pRT1957**	p426GPD-Nas2	This study
**pRT2214**	pET42b-Rpn3 : Sem1 : HA-Rpn7 : 6His-Rpn12(ΔBamHI)	This study
**pRT2226**	pCDF42b-MBP(ΔSacI)-3Cx-Rpn6(ΔEagI) : Rpn9 : Rpn11(ΔEagI) : Rpn5 : Rpn8	This study
**pRT2316**	pACYCDuet-1-tRNAs : Rpn14 : Nas6 : Nas2 : Hsm3	This study
**pRT2591**	pQE80LKan-14His-bdNEDD8-NbALFAST-Cys	This study
**pRT2600**	pSNR52-sgRNA/Cas9 pre8-K63 gRNA	This study
**pRT2617**	pETDuet-12His-bdSUMO-scHsm3	This study
**pRT2627**	p414GPD-Hsm3	This study
**pRT2635**	p414GPD-Nas2	This study

References

1. Park, S.; Roelofs, J.; Kim, W.; Robert, J.; Schmidt, M.; Gygi, S. P.; Finley, D., Hexameric assembly of the proteasomal ATPases is templated through their C termini. *Nature* **2009,** *459* (7248), 866-70.
2. Mumberg, D.; Müller, R.; Funk, M., Yeast vectors for the controlled expression of heterologous proteins in different genetic backgrounds. *Gene* **1995,** *156* (1), 119-22.
3. Gietz, R. D.; Sugino, A., New yeast-Escherichia coli shuttle vectors constructed with in vitro mutagenized yeast genes lacking six-base pair restriction sites. *Gene* **1988,** *74* (2), 527-34.
4. Beckwith, R.; Estrin, E.; Worden, E. J.; Martin, A., Reconstitution of the 26S proteasome reveals functional asymmetries in its AAA+ unfoldase. *Nature structural & molecular biology* **2013,** *20* (10), 1164-72.

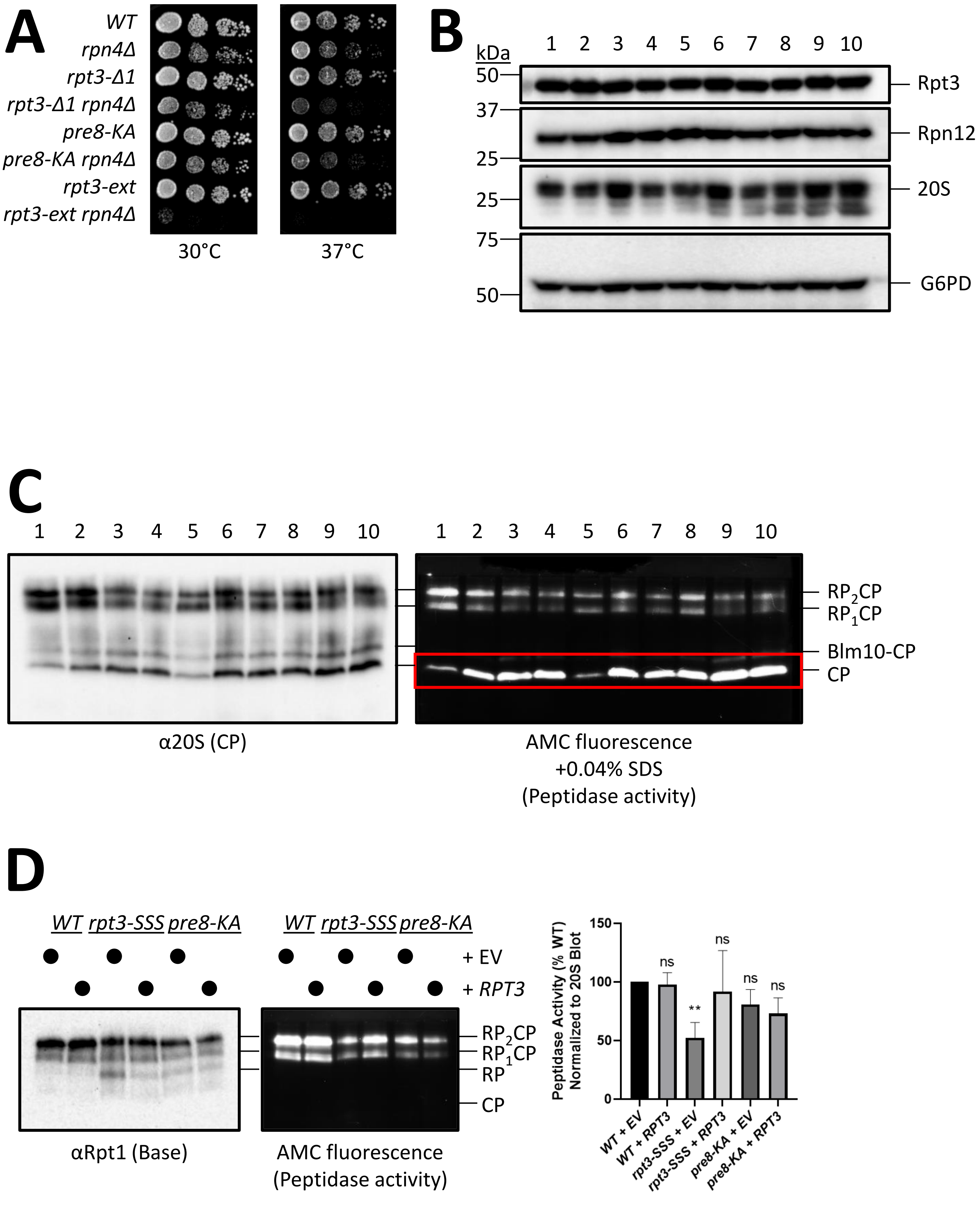

Warnock *et al,* Figure S1

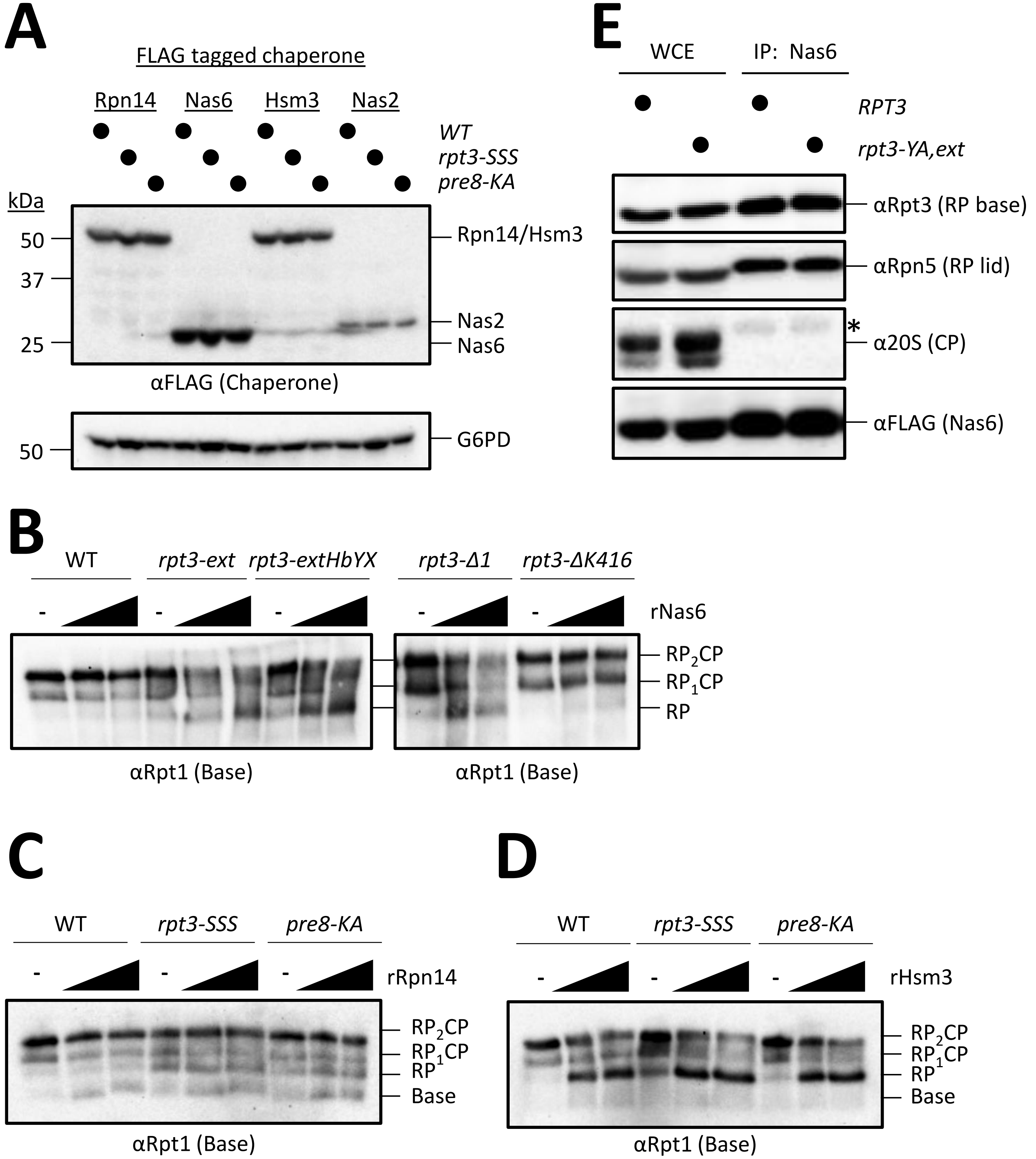

Warnock *et al,* Figure S2

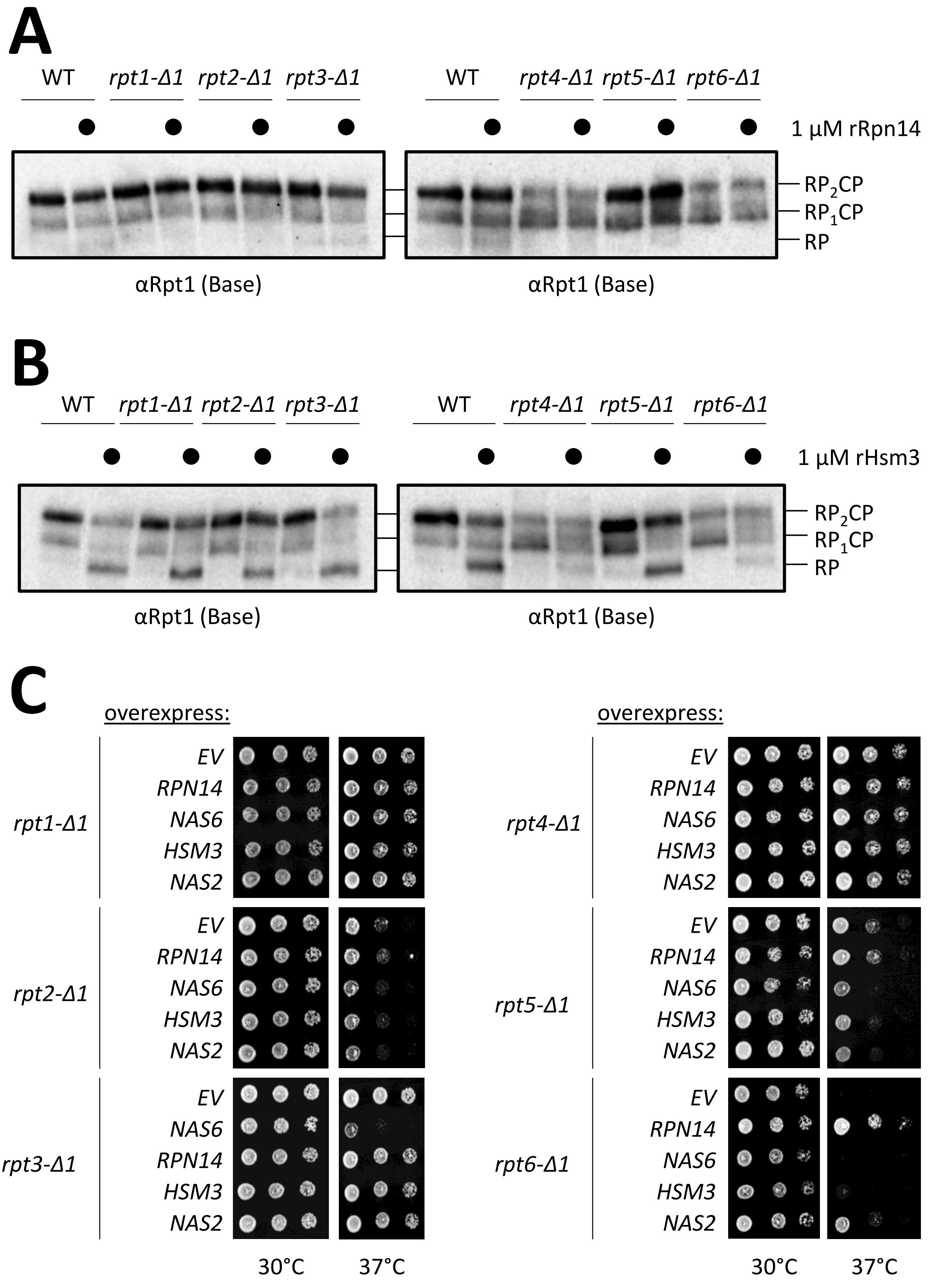

Warnock *et al,* Figure S3

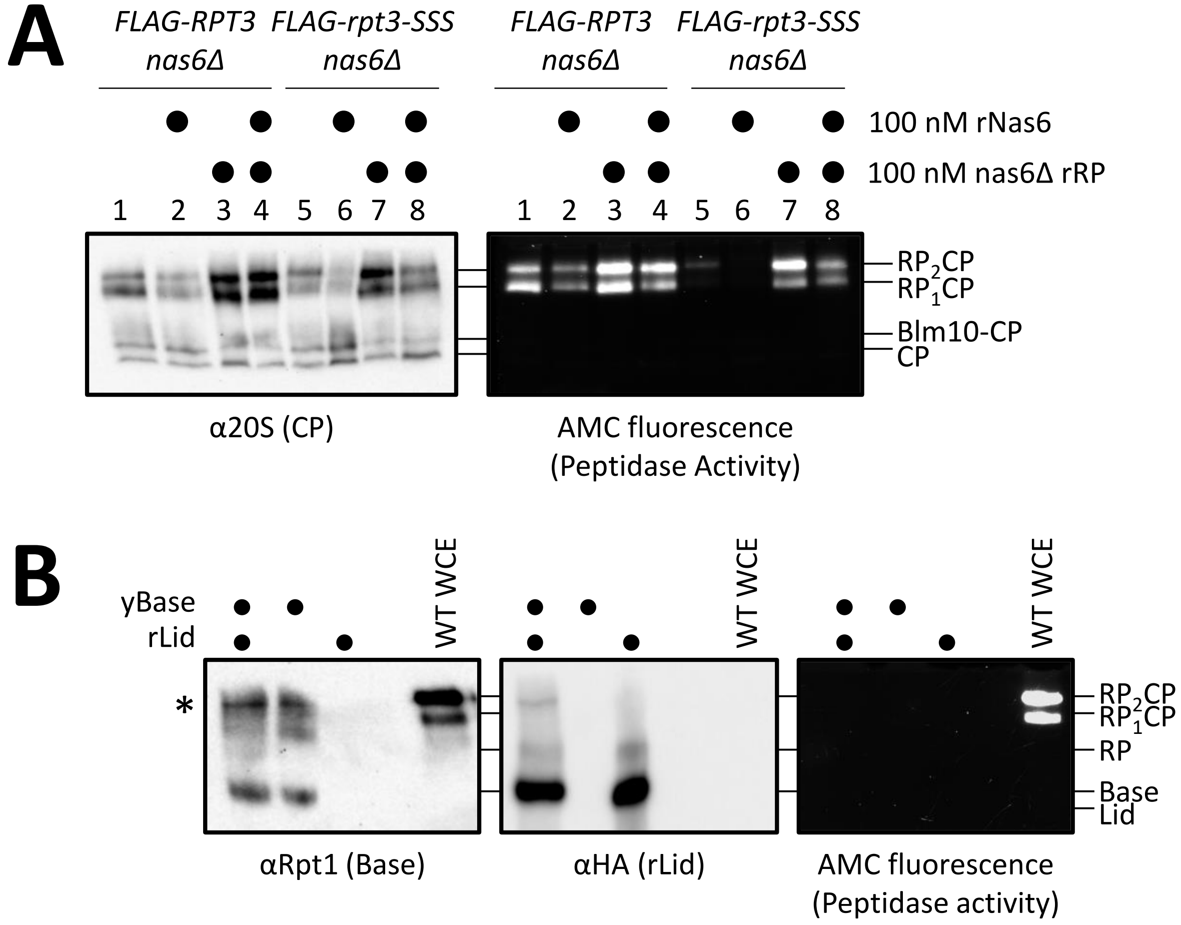
A
FLAG-RPT3 nas6Δ
FLAG-rpt3-SSS nas6Δ
FLAG-RPT3 nas6Δ
FLAG-rpt3-SSS nas6Δ
100 nM rNas6
100 nM nas6Δ rRP
1 2 3 4 5 6 7 8
1 2 3 4 5 6 7 8
RP2CP
RP1CP
Blm10-CP
CP
α20S (CP)
AMC fluorescence (Peptidase Activity)
B
yBase
rLid
WT WCE
WT WCE
WT WCE
*
RP2CP
RP1CP
RP
Base
Lid
αRpt1 (Base)
αHA (rLid)
AMC fluorescence (Peptidase activity)

Assembly chaperone Nas6 selectively destabilizes 26S proteasomes with defective regulatory particle-core particle interfaces

Jennifer L. Warnock, Gabriel W. Jobin, Sandhya Kumar, and Robert J. Tomko Jr.

Supporting Information: Supplemental Figure Legends

Supplemental Figure S1. **A)** Growth analyses of the indicated Rpt3 tail docking mutants alone and when combined with deletion of *RPN4*. Serial dilutions of the indicated strains were plated on YPD media and incubated at indicated temperatures for 2 days. **B)** Western blot analysis of Rpt3 C-terminal mutants (numbered as in **Fig. 2A**) showing overall levels of Rpt3, Rpn12, and 20S. Glucose-6-phosphate dehydrogenase (G6PD) is included as a loading control. **C)** Anti-20S immunoblot and in-gel peptidase assay of native PAGE-separated extracts of the Rpt3 tail docking mutant strains (numbered as in **Fig. 2A**). The in-gel peptidase assay was performed in the presence of SDS to quantify the activity of proteasomes upon opening of the CP gate and of the free CP (red box) present in the extracts. **D)** A WT copy of *RPT3* was expressed in WT, *rpt3-SSS*, and *pre8-KA* cells, with an empty vector (EV) used as a control. Native blot analysis and in-gel peptidase assay (quantification on right) showed that expression of WT *RPT3* rescued the peptidase activity defect present in *rpt3-SSS*, but not *pre8-KA* proteasomes. One-way ANOVA with Tukey's test for multiple comparisons; error bars represent ± SD; ns, not significant; **, $p < 0.01$; N=4.

Supplemental Figure S2. **A)** Western blot analysis of assembly chaperone expression in WT and two of the Rpt3 tail docking mutants from **Fig. 3B**. Chaperones were expressed as C-terminal 3xFLAG fusions from their native chromosomal loci. **B)** Native PAGE analysis of WT and Rpt3 C-terminal mutant extracts upon addition of different concentrations of rNas6 (0, 0.1, and 10 µM). **C-D)** Native PAGE analysis of WT and Rpt3 tail docking mutant extracts after addition of increasing amounts (0, 0.1, or 1.0 µM) of purified recombinant (r) Rpn14 (**C**) or Hsm3 (**D**). **E)** Nas6 does not co-purify the CP from *rpt3-ext* extracts. Nas6 was immunoprecipitated from the indicated whole cell extracts (WCE) via a C-terminal 3xFLAG tag. After elution of Nas6 and associated proteins with 3xFLAG peptide, the WCE and eluates were subjected to immunoblotting with antibodies against the indicated proteins. Asterisk, nonspecific band.

Supplemental Figure S3. **A-B)** Native PAGE analysis of each Rpt tail truncation mutant with the addition of rRpn14 (**A**) or rHsm3 (**B**). **C)** Growth assay analysis of tail truncation mutants overexpressing each of the RP assembly chaperones. Serial dilutions of the indicated strains transformed with pGPD416 plasmids overexpressing the listed chaperones were plated on SC-TRP media and incubated as shown for 2 days.

Supplemental Figure S4. **A)** Anti-20S blot and in-gel peptidase assay of the same samples as in **Fig. 6D**. **B)** Native PAGE analysis of purified *nas6Δ* yBase and rLid. Assembly of rRP mixture was conducted as described in **Fig. 6D**. Assembled rRP mixture was run alongside purified rLid and

nas6Δ yBase. The poor assembly efficiency *in vitro* likely arises from the absence of Nas6 and/or other factors normally present in cells. The asterisk indicates the position of an Rpt1-reactive species of unknown composition that lacks peptidase activity and migrates slightly faster than RP_2CP. This species is similar to one observed in other studies utilizing purified yeast base (Kleijnen *et al*. NSMB 2007).
